# Kernel Manifold Alignment for Domain Adaptation

**DOI:** 10.1371/journal.pone.0148655

**Published:** 2016-02-12

**Authors:** Devis Tuia, Gustau Camps-Valls

**Affiliations:** 1 MultiModal Remote Sensing, University of Zurich, Zurich, Switzerland; 2 Image Processing Laboratory, Universitat of València, València, Spain; Jiangnan University, CHINA

## Abstract

The wealth of sensory data coming from different modalities has opened numerous opportunities for data analysis. The data are of increasing volume, complexity and dimensionality, thus calling for new methodological innovations towards multimodal data processing. However, multimodal architectures must rely on models able to adapt to changes in the data distribution. Differences in the density functions can be due to changes in acquisition conditions (pose, illumination), sensors characteristics (number of channels, resolution) or different views (e.g. street level vs. aerial views of a same building). We call these different acquisition modes *domains*, and refer to the adaptation problem as *domain adaptation*. In this paper, instead of adapting the trained models themselves, we alternatively focus on finding mappings of the data sources into a common, semantically meaningful, representation domain. This field of *manifold alignment* extends traditional techniques in statistics such as canonical correlation analysis (CCA) to deal with nonlinear adaptation and possibly non-corresponding data pairs between the domains. We introduce a kernel method for manifold alignment (KEMA) that can match an arbitrary number of data sources without needing corresponding pairs, just few labeled examples in all domains. KEMA has interesting properties: 1) it generalizes other manifold alignment methods, 2) it can align manifolds of very different complexities, performing a discriminative alignment preserving each manifold inner structure, 3) it can define a domain-specific metric to cope with multimodal specificities, 4) it can align data spaces of different dimensionality, 5) it is robust to strong nonlinear feature deformations, and 6) it is closed-form invertible, which allows transfer across-domains and data synthesis. To authors’ knowledge this is the first method addressing all these important issues at once. We also present a reduced-rank version of KEMA for computational efficiency, and discuss the generalization performance of KEMA under Rademacher principles of stability. Aligning multimodal data with KEMA reports outstanding benefits when used as a data pre-conditioner step in the standard data analysis processing chain. KEMA exhibits very good performance over competing methods in synthetic controlled examples, visual object recognition and recognition of facial expressions tasks. KEMA is especially well-suited to deal with high-dimensional problems, such as images and videos, and under complicated distortions, twists and warpings of the data manifolds. A fully functional toolbox is available at https://github.com/dtuia/KEMA.git.

## Introduction

Domain adaptation constitutes a field of high interest in pattern analysis and machine learning. Classification algorithms developed with data from one domain cannot be directly used in another related domain, and hence adaptation of either the classifier or the data representation becomes strictly imperative [1]. For example, there is actually strong evidence that a significant degradation in the performance of state-of-the-art image classifiers is due to test domain shifts, such as changing image sensors and noise conditions [2], pose changes [3], consumer vs. commercial video [4], and, more generally, datasets biased due to changing acquisition procedures [5].

Adapting (modifying) the classifier for any new incoming situation requires either computationally demanding retraining, passive-aggressive strategies, online filtering, or sample-relevance estimation and weighting. These approaches are algorithm-dependent, often resort to heuristic parameters, require good estimates of sample relevance and information content. The ever-evolving classifier is also very hard to analyze. Alternatively, one may also try to adapt the domain representations to a single latent space, and then apply a unique single classifier in that *semantically meaningful* feature space. In this paper, we focus on the latter pathway. Adapting the representation space has been referred in the literature to as *feature representation transfer*[6] or *feature transformation learning*[7].

### Related works

The literature of feature representation transfer can be divided into three families of adaptation problems, depending on the availability of labels in the different domains. They are briefly reviewed hereafter and their main properties are summarized in Table 1. We discuss on the main the type of domain adaptation method (supervised, unsupervised or semisupervised), the capability to align several domains or possibly unpaired examples, and eventually of different dimensionality, and the linear or nonlinear nature of the transformation.

**Table 1:** Properties of feature representation transfer methods.

	DA type			Properties			
Method	Unsup.	Semis.	Sup.	*D* ≥ 2	Unpaired	*d*_*S*_ ≠ *d*_*T*_	Nonlinear
PCA [24]	√				√		
KPCA [25]	√				√		√
TCA [10]	√				√		√
SSTCA [10]		√			√		√
JDA [26]		√	$\hat{y}$		√		√
CCA [22]	√			√		√	
kCCA [9]	√			√		√	√
MA [20]			*p*	√			
GM [27]	√				√		
OT-lab [15]		√			√		√
SGF [12]	√	√	√		√		√
GFK [13]	√	√	√		√		√
MMDT [18]			√		√		
SSMA [23]			√	√	√	√	
KEMA			√	√	√	√	√

*D*: number of domains.

*d*_*S*_, *d*_*T*_: number of features in source and target.

$\hat{y}$: semilabels predicted by a classifier.

*p*: known corresponding samples, but no labels.

#### Unsupervised adaptation

First attempts of unsupervised domain adaptation are found in multiview analysis [8], and more precisely in canonical correlation analysis (CCA) and kernel CCA (KCCA) [9]. Despite their good performance in general, they still require points in different sources to be corresponding pairs, which is often hard to meet in real applications. Think, for example, of exploiting images, text and video in Wikipedia for document categorization, trying to align images with different geometrical resolutions containing similar (not necessarily the same) objects, or comparing commercial product images with consumer snapshots of the same product. These real applications seldom provide datasets with corresponding pairs and/or features. Alternative methods seek for a set of projectors that minimize a measure of discrepancy between the source and target data distributions, such as the Maximum Mean Discrepancy (MMD) [10] or the recent geodesic distance between distributions [11]. However, to compare distributions, the data are supposed to be represented by the same features in all domains. The idea of exploiting geodesic distances along manifolds was also considered in [12], where a finite set of intermediate transformed data distributions are sampled along the geodesic flow (SGF) between the linear subspaces. The intermediate features are then used to train the classifier. The idea was extended in [13], where a Geodesic Flow Kernel (GFK) was constructed by considering the infinity of transformed subspaces along the geodesic path. However, both SGF and GFK assume input data space of the same dimensionality.

#### Semi-supervised adaptation with labels in the source domain only

A second family of methods exploits the wealth of unsupervised information along with the limited amount of labeled data in the source domain to guide the adaptation. Actually, some of the above-mentioned methods can incorporate the information of labeled samples in the source domain: the Transfer Component Analysis [10] becomes semi-supervised by maximizing the Hilbert-Schmidt Independence Criterion (HSIC) [14] between a kernel on features and a kernel on labels in the source domain, while SGF [12] and GFK [13] become semi-supervised if the eigenvectors of the source domain are found with a discriminative feature extractor such as partial least squares (PLS). Another family of methods, collectively known as Optimal Transport (OT) techniques, can use labeled samples in the source domain to maximize coherence in the transportation plan of masses between source and target domains [15]. For this last method, the transformation is defined such that the transformed source distribution has ideally the same probability density as the target one, and simultaneously the labeled examples in the source domain remain grouped together.

#### Supervised adaptation with labels in all domains

SGF and GFK can be also defined for the case in which all the domains are labeled. Saenko et al. [2] learned transformations between the domains as a dot product between the (linearly) transformed source samples. The method was extended in [16] to domains of different dimensionality, and in [17] to problems with multiple domains. Alternative approaches try to align target and source features while simultaneously moving labeled examples to the correct side of a decision hyperplane (MMDT) [18]. Donahue et al. extended this reasoning by including Laplacian regularization [19]. A last family of supervised methods is known as *manifold alignment*, and aims at concurrently matching the corresponding instances while preserving the topology of each input domain, generally using a graph Laplacian [20, 21]. Roughly speaking, aligning data manifolds reduces to finding projections to a common latent space where all datasets show similar statistical characteristics. Manifold alignment (MA) is a new form of multivariate analysis that dates back to the work of Hotelling in 1936 on canonical correlation analysis (CCA) [22], where projections try to correlate the data sources onto a common target domain. While appealing, these methods still require specifying a small amount of cross-domain sample correspondences. The problem was addressed in [23] by relaxing the constraint of paired correspondences with the constraint of having the same class labels in all domains. The semi-supervised manifold alignment (SSMA) method proposed in [23] projects data from different domains to a latent space where samples belonging to the same class become closer, those of different classes are pushed far apart, and the geometry of each domain is preserved. The method performs well in general and can deal with multiple domains of different dimensionality. However, SSMA cannot cope with strong nonlinear deformations and high-dimensional data problems.

### Contributions

This paper introduces a generalization of SSMA through kernelization for manifold alignment and domain adaptation. The proposed Kernel Manifold Alignment (KEMA) has some remarkable appealing properties:

1. KEMA generalizes other manifold alignment methods. Being a kernel method, KEMA reduces to SSMA [23] when using a linear kernel, thus allowing to deal with high-dimensional data efficiently in the dual form (*Q*-mode analysis): therefore KEMA can cope with input space of very large dimension, e.g. extracted by Fisher vectors or deep features. KEMA also generalizes other manifold alignment methods, e.g. [20] when used with a linear kernel and with sample correspondences instead of the class similarity matrices (see page 5);
2. KEMA goes beyond data rotations and can align manifolds of very different structure, performing a flexible discriminative alignment that preserves the manifold structure;
3. KEMA defines a domain-specific metric when using different kernel functions in the different domains. Contrarily to SSMA, KEMA can use different kernels in each domain, thus allowing to use the best descriptor for each data source at hand, e.g. when aligning text and images one could involve using (more appropriate) string or histogram kernels in the very same alignment procedure, or using the same kernel function with different hyperparameters in each domain;
4. As SSMA, KEMA can align data spaces of different dimensionality. This is an advantage with respect to other feature representation transfer approaches that require either sample correspondences [9, 12, 15, 20] or strict equivalence of the feature spaces across domains [2, 10, 25].
5. KEMA is robust to strong (nonlinear) deformations of the manifolds to be aligned, as the kernel compensates for problems in graph estimation and numerical problems. As noted above, the use of different metric stemming from different kernels reinforces the flexibility of the approach;
6. Mapping data between domains (and hence data synthesis) can be performed in closed-form, thus allowing to measure the quality of the alignment in physical units. Kernelization typically makes the new method not invertible *analytically*, and one commonly resorts to approximate methods for estimating pre-images [28–30]. For the case of KEMA, this is not straightforwad (see page 8). As an alternative, we propose a chain of transforms of different types as a simple, yet efficient way of performing the inversion accurately and in closed form.

The reported theoretical advantages translate into outstanding convenience when working with high-dimensional problems and strong distortions in the manifold structures, as illustrated on a large set of synthetic and real applications in the experimental section.

## Materials and Methods

In this section, we first recall the linear SSMA algorithm and then derive our proposed KEMA. We discuss its theoretical properties, the stability bounds and propose a reduced rank algorithm, as well as a closed-form inversion strategy.

### Semi-supervised manifold alignment

Semi-supervised learning consists in developing inference models that collectively incorporate labeled and unlabeled data in the model definition. In semi-supervised learning (SSL) [31], the algorithm is provided with some available *labeled* information in addition to the *unlabeled* information, thus allowing to encode some knowledge about the geometry and the shape of the dataset. There is an overwhelming amount of SSL methods in the literature, yet the vast majority of algorithms try to encode the relations between labeled and unlabeled data through the definition of an undirected graph, and more precisely through the graph Laplacian matrix **L**.

To define **L**, let’s first define a graph *G*(*V*, *E*) with a set of *n* nodes, *V*, connected by a set of edges, *E*. The edge connecting nodes *i* and *j* has an associated weight [31]. In this framework, the nodes are the samples, and the edges represent the similarity among samples in the dataset. A proper definition of the graph is the key to accurately introduce data structure in the model.

To understand how matrix **L** is constructed, two mathematical tools have to be introduced [31, 32]: First, the *adjacency* matrix **W**, which contains the neighborhood relations between samples. It has non-zero entries only between neighboring samples, which are generally found by *k*-nearest neighbors or an *ϵ*-ball distance. Then, the *degree* matrix **D**, which is a diagonal matrix of size *n* × *n* containing the number of connections to a node (degree). The Laplacian matrix **L** is then defined as **L** = **D** − **W**. Intuitively, **L** measures the variation (i.e. norm of derivatives hence the name of Laplacian operator) of the decision function along the graph built upon all (labeled and unlabeled) samples [31].

**Fig 1:**
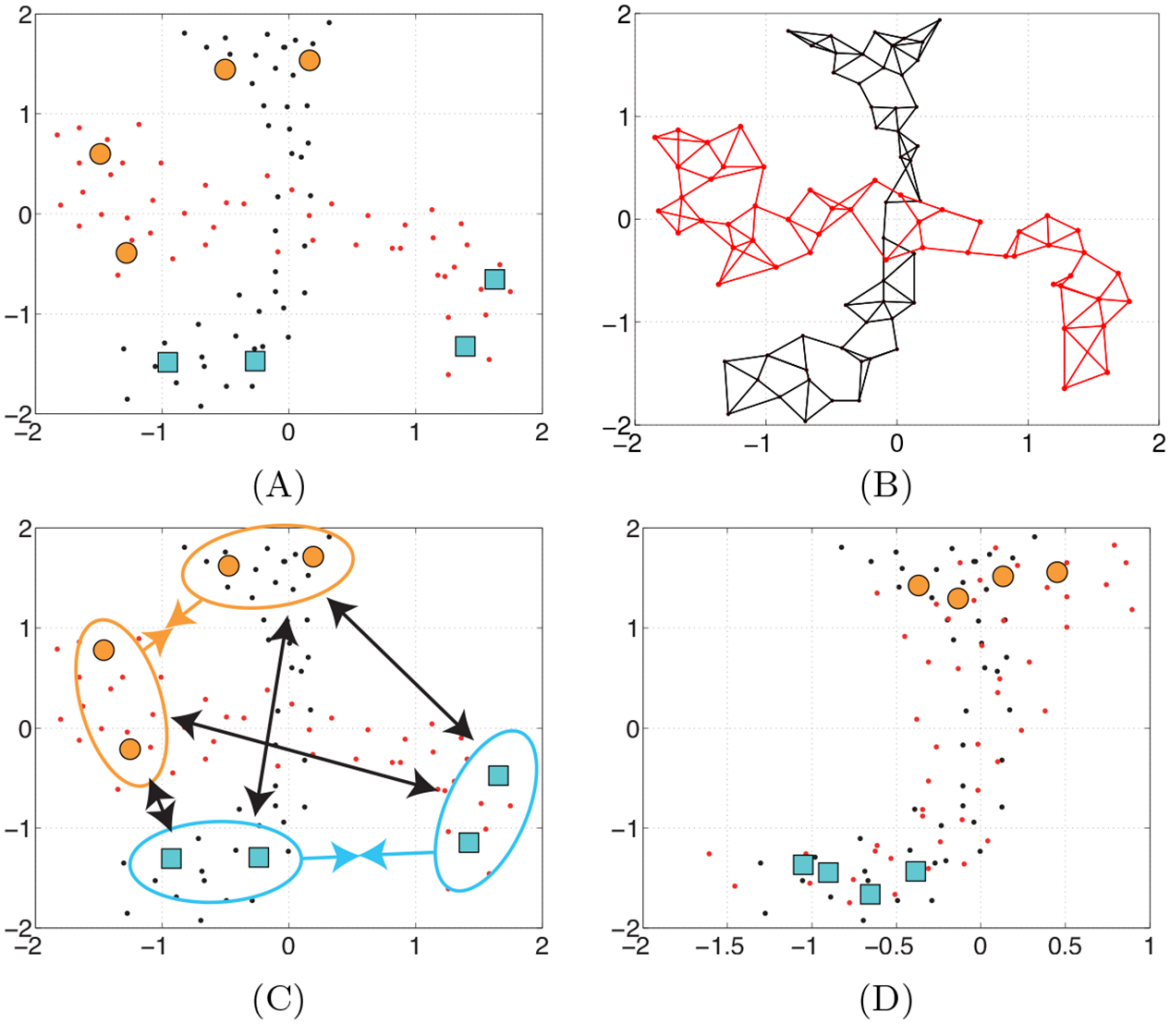
The idea behind semi-supervised manifold alignment. (A) Consider two data sources (red and black small points) in a binary problem (labeled points in orange balls and blue squares). SSMA aligns the dataset by (B) preserving their inner geometry and (C) registering the data clouds in the feature space using labels. (D) After alignment the datasets live in a semantically meaningful space.

When it comes to manifold alignment, an interesting semisupervised approximation was presented in [23]. Let us consider *D* domains $X_{i}$ representing similar classification problems. The corresponding data matrices, $X_{i}\in R^{d_{i}\times n_{i}}$, *i* = 1, …, *D*, contain *n*_*i*_ examples (labeled, *l*_*i*_, and unlabeled, *u*_*i*_, with *n*_*i*_ = *l*_*i*_+*u*_*i*_) of dimension *d*_*i*_, and $n=\sum_{i=1}^{D}n_{i}$. The SSMA method [23] maps all the data to a latent space F such that samples belonging to the same class become closer, those of different classes are pushed far apart, and the geometry of the data manifolds is preserved. Therefore, three entities have to be considered, leading to three *n* × *n* matrices: 1) a similarity matrix **W**_*s*_ that has components $W_{s}^{ij}=1$ if **x**_*i*_ and **x**_*j*_ belong to the same class, and 0 otherwise (including unlabeled); 2) a dissimilarity matrix **W**_*d*_, which has entries $W_{d}^{ij}=1$ if **x**_*i*_ and **x**_*j*_ belong to different classes, and 0 otherwise (including unlabeled); and 3) a similarity matrix that represents the topology of a given domain, **W**, e.g. a radial basis function (RBF) kernel or a *k* nearest neighbors graph computed for each domain separately and joined in a block-diagonal matrix. Since we are not interested in preserving geometrical similarity between the domains (we are only interested in preserving their inner geometry), all the elements of the off-diagonal blocks in the matrix **W** are zeros. On the contrary, **W**_*s*_ and **W**_*d*_ are defined between the domains and therefore act as registration anchor points in the feature space. An illustrative example of how SSMA works in given in Fig 1. The three different entities lead to three different graph Laplacians: **L**_*s*_, **L**_*d*_, and **L**, respectively. Then, the SSMA embedding must minimize a joint cost function essentially given by the eigenvectors corresponding to the smallest non-zero eigenvalues of the following generalized eigenvalue problem:

$$Z\left(L+\mu L_{s}\right)Z^{⊤}V=\lambda ZL_{d}Z^{⊤}V,$$ (1)

where **Z** is a block diagonal matrix containing the data matrices **X**_*i*_, **Z** = diag(**X**_1_, ⋯,**X**_*D*_), and **V** contains in the columns the eigenvectors organized in rows for the particular domain, **V** = [**v**_1_,**v**_2_, …,**v**_*D*_]^⊤^, see details in [21, 33]. The method allows to extract a maximum of $N_{f}=\sum_{i=1}^{D}d_{i}$ features that serve for projecting the data to the common latent domain as follows:

$$P_{F}\left(X_{i}\right)=v_{i}^{⊤}X_{i}.$$ (2)

Advantageously, SSMA can easily project data between domains *j* and *i*: first mapping the data in $X_{j}$ to the latent domain F, and from there inverting back to the target domain $X_{i}$ as follows:

$$P_{i}\left(X_{j}\right)={\left(v_{j}v_{i}^{†}\right)}^{⊤}X_{j},$$ (3)

where ^†^ represents the pseudo-inverse of the eigenvectors of the target domain. The operation is depicted as: 
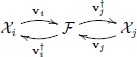
 Therefore, the method can be used for domain adaptation but also for data synthesis. This property was pointed out in [23], and experimentally studied for image analysis in [34].

### Kernel manifold alignment

When using linear algorithms, a well-established theory and efficient methods are often available. Kernel methods exploit this fact by embedding the data set *S* defined over the input or attribute space X(S⊆X) into a higher (possibly infinite) dimensional Hilbert space H, or *feature space*, and then they build a linear algorithm therein, resulting in an algorithm which is nonlinear with respect to the input data space. The mapping function is denoted as ϕ:X→H. Though linear algorithms will benefit from this mapping because of the higher dimensionality of the feature space, the computational load would dramatically increase because we should compute sample coordinates in that high dimensional space. This computation is avoided through the use of the kernel trick by which, if an algorithm can be expressed with dot products in the input space, its (nonlinear) kernel version only needs the dot products among mapped samples. Kernel methods compute the similarity between training samples $S={\left\{x_{i}\right\}}_{i=1}^{n}$ using pair-wise inner products between mapped samples, and thus the so-called kernel matrix **K**_*ij*_ = *K*(**x**_*i*_, **x**_*j*_) = 〈*ϕ*(**x**_*i*_), *ϕ*(**x**_*j*_)〉 contains all the necessary information to perform many classical linear algorithms in the feature space.

#### Kernelization of SSMA

Kernelization of SSMA is apparently straightforward; one should map the data to a Hilbert feature space and then replace all instances of dot products with kernel functions. However, note that in the original formulation of SSMA, there are *D* data sources that need to be first mapped to a common feature space. For doing this, we need to define *D* different feature mappings to eventually different Hilbert feature spaces, and then ensure that mapped data live in the same subspace in order to do linear operations therein with *all* mapped data sources. This can be actually done by resorting to a property of Functional Analysis Theory [35], the *direct sum of Hilbert spaces*.

**Theorem 1** **Direct sum of Hilbert spaces [35]:** *Given two Hilbert spaces*, $H_{1}$ *and* $H_{2}$, *the set of pairs* {**x**,**y**} *with* $x\in H_{1}$ *and* $y\in H_{2}$ *is a Hilbert space* H *with inner product* $\langle \left\{x_{1},y_{1}\right\},\left\{x_{2},y_{2}\right\}\rangle ={\langle x_{1},x_{2}\rangle }_{H_{2}}+{\langle y_{1},y_{2}\rangle }_{H_{2}}$. *This is called the direct sum of the spaces, and is denoted as* $H=H_{1}⊕H_{2}$. *This property extends to a finite summation of* *D* *Hilbert spaces by which* $H={⊕}_{i=1}^{D}H_{i}$ *is a Hilbert space*.

Now we have the necessary tools for kernelizing the SSMA algorithm. Let us first map the *D* different datasets to *D* possibly different Hilbert spaces $H_{i}$ of dimension *H*_*i*_, $\phi_{i}\left(\cdot \right):x\mapsto \phi_{i}\left(x\right)\in H_{i}$, *i* = 1, …, *D*. Now, by replacing all the samples with their mapped feature vectors, the problem becomes:

$$\Phi \left(L+\mu L_{s}\right)\Phi^{⊤}U=\lambda \Phi L_{d}\Phi^{⊤}U,$$ (4)

where **Φ** is a block diagonal matrix containing the data matrices **Φ**_*i*_ = [*ϕ*_*i*_(**x**_1_), …, *ϕ*_*i*_(**x**_*n*_*i*__)]^⊤^ and **U** contains the eigenvectors organized in rows for the particular domain defined in Hilbert space $H_{i}$, **U** = [**u**_1_,**u**_2_, …,**u**_*H*_]^⊤^ where $H=\sum_{i}^{D}H_{i}$. Note that the eigenvectors **u**_*i*_ are of possibly infinite dimension and cannot be explicitly computed. Instead, we resort to the definition of *D* corresponding Riesz representation theorems [36] so the eigenvectors can be expressed as a linear combination of mapped samples [37], **u**_*i*_ = **Φ**_*i*_ *α*_*i*_, and in matrix notation **U** = **ΦΛ**. This leads to the problem:

$$\Phi \left(L+\mu L_{s}\right)\Phi^{⊤}\Phi \Lambda =\lambda \Phi L_{d}\Phi^{⊤}\Phi \Lambda .$$ (5)

Now, by pre-multiplying both sides by **Φ**^⊤^ and replacing the dot products with the corresponding kernel matrices, $K_{i}=\Phi_{i}^{⊤}\Phi_{i}$, we obtain the final solution:

$$K\left(L+\mu L_{s}\right)K\Lambda =\lambda KL_{d}K\Lambda ,$$ (6)

where **K** is a block diagonal matrix containing the kernel matrices **K**_*i*_. Now the eigenproblem becomes of size *n* × *n* instead of *d* × *d*, and we can extract a maximum of *N*_*f*_ = *n* features.

When a linear kernel is used for all the domains, $K_{i}=X_{i}^{⊤}X_{i}$, KEMA reduces to SSMA:

$$P_{F}\left(X_{i}\right)=\alpha_{i}^{⊤}X_{i}^{⊤}X_{i}={\left(X_{i}\alpha_{i}\right)}^{⊤}X_{i}=v_{i}^{⊤}X_{i}.$$ (7)

This dual formulation is advantageous when dealing with very high dimensional datasets, *d*_*i*_ ≫ *n*_*i*_ for which the SSMA problem is not well-conditioned. Operating in *Q*-mode endorses the method with numerical stability and computational efficiency in current high-dimensional problems, e.g. when using Fisher vectors or deep features for data representation. This type of problems with much more dimensions than points are recurrent nowadays for example in the fields of bioinformatics, chemometrics, and image and video processing. In this sense, even KEMA with a linear kernel becomes a valid solution for these problems, as it has all the advantages of CCA-like methods, but can also deal with unpaired data.

Projection to the latent space requires first mapping the data **X**_*i*_ to its corresponding Hilbert space $H_{i}$, thus leading to the mapped data **Φ**_*i*_, and then applying the projection vector **u**_*i*_ defined therein:

$$P_{F}\left(X_{i}\right)=u_{i}^{⊤}\Phi_{i}=\alpha_{i}^{⊤}\Phi_{i}^{⊤}\Phi_{i}=\alpha_{i}^{⊤}K_{i}.$$ (8)

which can be depicted as: 
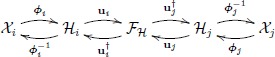
 Therefore, projection to the kernel latent space is possible through the use of dedicated reproducing kernel functions.

In order to map data from domain $X_{j}$ to domain $X_{i}$ with KEMA we would need to estimate *D* − 1 inverse mappings from the latent space to the corresponding target domain $X_{i}$. Such transformations are highly desirable in order to measure the accuracy of the alignment/adaptation in meaningful physical units. In general, nevertheless, using kernel functions hampers the invertibility of the transformation. One can show that if an exact pre-image exists, and if the kernel can be written as $k\left(x,x^{\prime }\right)=\psi_{k}\left(x^{⊤}x^{\prime }\right)$ with an invertible function *ψ*_*k*_(⋅), then one can compute the pre-image analytically under mild assumptions. However, it is seldom the case that exact pre-images exist, and one resorts to approximate methods such as those in [28–30]. In the case of KEMA, inversion from the latent space to the target domain $X_{i}$ is even harder, and hampers the use of standard pre-imaging techniques. Standard pre-image methods in kernel machines [28–30] typically assume a particular kernel method (e.g. kPCA) endorsed with a particular kernel function (often the polynomial or the squared exponential). If other kernel functions are used, the formulation should be derived again. Remember that our KEMA feature vectors in the latent space were obtained using a complex (and supervised) function that considers labeled and unlabeled samples from all available domains through the composition of kernel functions and graph Laplacians. One could derive the equations for preimaging under our eigenproblem setting, $K^{\prime }≔K_{s}^{-1}K_{d}$ where **K**_*s*_ ≔ **K**(**L** + *μ* **L**_*s*_)**K** and **K**_*d*_ = **KL**_*d*_ **K**, but this is very complicated, data dependent, and sensitive because of the appearance of several hyperparameters. Another alternative could be performing a sort of multidimensional regression (from the latent space to $X_{i}$) in a similar way to the kernel dependency estimation (KDE) method revised in [29], but the approach would be complicated (no guarantees about the existence of a kernel trying to reproduce the inverse mapping implicit in **K**′ exist), computationally demanding (many hyperparameters appear), and would not deliver a closed-form solution.

Here we propose a simple alternative solution to the mapping inversion: to use a linear kernel for the latent-to-target transformation $K_{i}=X_{i}^{⊤}X_{i}$, and **K**_*j*_ for *j* ≠ *i* with any desired form. Following this intuition, projection of data **X**_*j*_ to the target domain *i* becomes:

$$P_{i}\left(X_{j}\right)={\left(u_{i}^{†}\right)}^{⊤}\alpha_{j}^{⊤}K_{j}={\left(\alpha_{j}{\left(X_{i}\alpha_{i}\right)}^{†}\right)}^{⊤}K_{j},$$ (9)

where for the target domain we used **u**_*i*_ = **Φ**_*i*_ ***α***_*i*_ = **X**_*i*_ ***α***_*i*_. We should note that the solution is not unique since *D* different inverse solutions can be obtained depending on the selected target domain. Using different transforms to perform model inversion was also recently studied in [38]: here, instead of using an alternate scheme, we perform direct inversion by chaining different transforms, leading to an efficient closed-form solution. Such a simple idea yields impressive results in practice (see the experimental section, page 14).

## Computational efficiency and stability of KEMA

One of the main shortcomings of KEMA is related to the computational cost since two *n* × *n* kernel matrices are involved, being $n=\sum_{i=1}^{D}n_{i}$. KEMA complexity scales quadratically with *n* in terms of memory, and cubically with respect to the computation time. Also projection for new data requires the evaluation of *n* kernel functions *per* example, becoming computationally expensive for large *n*. To alleviate this problem, we propose two alternatives to speed up KEMA: a reduced-rank approximation (REKEMA) and a randomized features approximation (rKEMA). We compare both approaches in CPU time, and for rKEMA we study the convergence bound in *ℓ*_2_-norm based on matrix Bernstein inequalities. Finally, we study the stability of the obtained solution when solving a (regularized) generalized eigenproblem using a finite number of samples based on Rademacher principles.

### Reduced rank approximation

**Fig 2:**
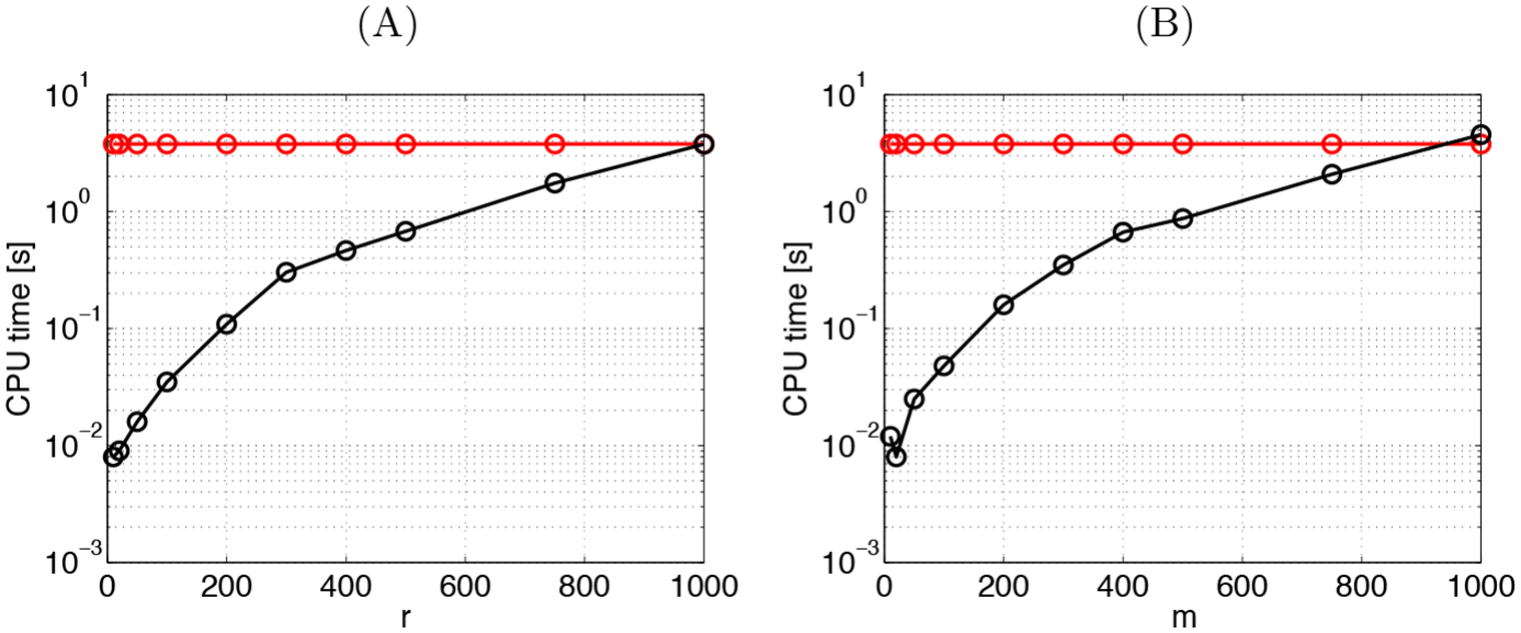
Average computational cost of REKEMA and *r*KEMA. CPU time [s], over 10 realizations as a function of *r* and *m* for the (A) reduced rank KEMA (REKEMA) and (B) randomized KEMA (rKEMA) in black lines. In both figures, the red line is the KEMA solution). We used synthetic example ♯1 (see experiments section) with *n* = 1000 samples.

The so-called reduced-rank Kernel Manifold Alignment (REKEMA) formulation imposes reduced-rank solutions for the projection vectors, **W** = **Φ**_*r*_ Λ, where **Φ**_*r*_ is a subset of the training data containing *r* samples (*r* ≪ *n*) and Λ is the new argument for the maximization problem. Plugging **W** into Eq (5), and replacing the dot products with the corresponding kernels, $K_{rn}=\Phi_{r}^{⊤}\Phi$, we obtain the final solution:

$$K_{rn}\left(L+\mu L_{s}\right)K_{nr}\Lambda =\lambda K_{rn}L_{d}K_{nr}\Lambda ,$$ (10)

where **K**_*rn*_ is a block diagonal matrix containing the kernel matrices **K**_*i*_ comparing a reduced set of *r* representative vectors and *all* training data points, *n*. REKEMA reports clear benefits for obtaining the projection vectors (the eigenproblem becomes of size *r* × *r* instead of *n* × *n*), hence the computational cost becomes $O(r^{3})$, *r* ≪ *n*, compacting the solution (now *N*_*f*_ = *r* ≪ *n* features), and in storage requirements (hence $O(r^{2})$). We want to highlight here that this is not a simple subsampling, because the model considers correlations between all training data and the reduced subset through **K**_*rn*_. The selection of the *r* points can be done in different ways and degrees of sophistication: close to centroids provided by a pre-clustering stage, extremes of the convex hull, sampling to minimize the reconstruction error or preserve information, form compact basis in feature space, etc. While such strategies are crucial in low-to-moderate sample-size regimes, random selection offers an easy way to select the *r* points and is the most widely used strategy. Fig 2A shows the evolution of the computational cost as a function of (randomly selected) *r* samples in a toy example of aligning two spirals (cf. experiment ♯1 in the experiments section).

### Random features approximation

A recent alternative to reduced rank approximations exploits the classical Bochner’s theorem in harmonic analysis, which has been recently introduced in the field of kernel methods [39]. The Bochner’s theorem states that a continuous kernel *k*(**x**,**y**) = *k*(**x** − **y**) on $R^{d}$ is positive definite (p.d.) if and only if *k* is the Fourier transform of a non-negative measure. If a shift-invariant kernel *k* is properly scaled, its Fourier transform *p*(**w**) is a proper probability distribution. This property is used to approximate kernel functions and matrices with linear projections on *m* random features as follows:

$$\begin{array}{cc} k\left(x,y\right) & =\int_{R^{d}}p\left(w\right)e^{-jw^{⊤}\left(x-y\right)}dw\approx \sum_{i=1}^{m}\frac{1}{m}e^{-jw_{i}^{⊤}x}e^{jw_{i}^{⊤}y}\\ & =\sum_{i=1}^{m}\frac{1}{m}\cos\left(w_{i}^{⊤}x+b_{i}\right)\cos\left(w_{i}^{⊤}y+b_{i}\right)=\left\langle \frac{1}{\sqrt{m}}z\left(x\right),\;\frac{1}{\sqrt{m}}z\left(y\right)\right\rangle , \end{array}$$ (11)

where *p*(**w**) is set to be the inverse Fourier transform of *k* and $b_{i}∼U\left(0,2\pi \right)$[39]. Therefore, we can randomly sample parameters $w_{i}\in R^{d}$ from a data-independent distribution *p*(**w**) and construct a *m*-dimensional randomized feature map **z**(⋅): **X** → **Z**, for data $X\in R^{n\times d}$ and $Z\in R^{n\times m}$, as follows:

$$\begin{array}{ll} & w_{1},\ldots ,w_{m}∼p\left(w\right),\\ z_{i} & ≔\left[\text{cos}\left(w_{i}^{⊤}x_{1}+b_{i}\right),\ldots ,\text{cos}\left(w_{i}^{⊤}x_{n}+b_{i}\right)\right]\in {\mathbb{R}}^{n},\\ z\left(X\right) & ≔Z=\left[z_{1}\cdots z_{m}\right]\in {\mathbb{R}}^{n\times m}. \end{array}$$ (12)

For a collection of *n* data points, ${\left\{x_{i}\right\}}_{i=1}^{n}$, a kernel matrix $K\in R^{n\times n}$ can be approximated with the explicitly mapped data, $Z\in R^{n\times m}$, $\hat{K}\approx ZZ^{⊤}$. The Gaussian kernel $k\left(x,y\right)=\exp(-\|x-y{\|}_{2}^{2}/\left(2\sigma^{2}\right))$ can be approximated using $w_{i}∼N\left(0,I/\sigma^{2}\right)$. For the case of KEMA, we have to sample twice, hence obtain two sets of vectors and associated matrices **Z**_*s*_ and **Z**_*d*_, to approximate the similarity and dissimilarity kernel matrices, $K_{s}≔K\left(L+\mu L_{s}\right)K\approx Z_{s}Z_{s}^{⊤}$ and $K_{d}≔KL_{d}K\approx Z_{d}Z_{d}^{⊤}$. The associated cost by using the random features approximation now reduces to $O(nm^{2})$, see Fig 2B. It is also important to notice that solving the generalized eigenvalue problem in KEMA feature extraction with random features converges in *ℓ*_2_-norm error with $O(m^{-1/2})$ and logarithmically in the number of samples when using an appropriate random parameter sampling distribution [40] (see the Appendix).

### Stability of KEMA

The use of KEMA in practice raises, however, the important question of the amount of data needed to provide an accurate empirical estimate, and how the quality of the solution differs depending on the datasets. Such results have been previously derived for KPCA [41] and KPLS [42] and here we extend them to our generalized eigenproblem setting. We focus on the concentration of sums of eigenvalues of the generalized KEMA eigenproblem solved using a finite number of samples, where new points are projected into the *m*-dimensional space spanned by the *m* eigenvectors corresponding to the largest *m* eigenvalues.

Following the notation in [41], we refer to the projection onto a subspace *U* of the eigenvectors of our eigenproblem as *P_U_*(*ϕ*(**x**)). We represent the projection onto the orthogonal complement of *U* by *P*_U^⊥^_(*ϕ*(**x**)). The norm of the orthogonal projection is also referred to as the residual since it corresponds to the distance between the points and their projections.

**Theorem 2 (Th. 1 and 2 in [41])** *Let us define* **K**_*s*_ ≔ **K**(**L** + *μ* **L**_*s*_)**K** *and* **K**_*d*_ = **KL**_*d*_ **K**. *If we perform KEMA in the feature space defined by* $K^{*}≔K_{s}^{-1}K_{d}$, *then with probability greater than* 1 − *δ* *over* *n* *random samples* *S*, *for all* 1 ≤ *r* ≤ *n*, *if we project data on the space* ${\hat{U}}_{r}$, *the expected squared residual is bounded by*

$$\sum_{j=r+1}^{n}\lambda_{j}\leq E\left[{\|P_{{{\hat{U}}_{r}}^{⊥}}\|}^{2}\right]\leq \min_{1\leq l\leq r}\left[\frac{1}{n}\sum_{j=l+1}^{n}{\hat{\lambda }}_{j}\left(S\right)+\frac{1+\sqrt{l}}{\sqrt{n}}\sqrt{\frac{2}{n}\sum_{i=1}^{n}K_{ii}^{*2}}\right]+R^{2}\sqrt{\frac{18}{n}\ln\left(\frac{2n}{\delta }\right)}$$ (13)

*and*

$$\sum_{j=1}^{r}\lambda_{j}\leq E\left[{\|P_{{\hat{U}}_{r}}\|}^{2}\right]\leq \max_{1\leq l\leq r}\left[\frac{1}{n}\sum_{j=1}^{l}{\hat{\lambda }}_{j}\left(S\right)-\frac{1+\sqrt{l}}{\sqrt{n}}\sqrt{\frac{2}{n}\sum_{i=1}^{n}K_{ii}^{*2}}\right]-R^{2}\sqrt{\frac{19}{n}\ln\left(\frac{2(n+1)}{\delta }\right)},$$ (14)

*where the support of the distribution is in a ball of radius* *R* *in the feature space and* *λ*_*i*_ *are* ${\hat{\lambda }}_{i}$ *are the process and empirical eigenvalues, respectively*.

**Theorem 3 (Regularized KEMA)** *The previous theorem holds only when the inverse* $K_{s}^{-1}$ *exists. Otherwise, we typically resort to matrix conditioning via regularization. Among the many possibilities in problem conditioning, the standard direct Tikhonov-Arnoldi approach helps solving the generalized eigenproblem on a shifted and inverted matrix, which damps the eigenvalues. Now we aim to bound a well-conditioned matrix* **K′** ≔ (**K**_*s*_ + γ**K**_*d*_)^−1^ **K**_*d*_, *where* *γ* > 0 *is the regularization parameter. It is easy to show that its estimated eigenvalues*, ${\hat{\theta }}_{i}$ *are related to the unregularized ones as* ${\hat{\lambda }}_{j}={\hat{\theta }}_{j}/\left(1-\gamma {\hat{\theta }}_{j}\right)$. *Therefore, with probability greater than* 1 − *δ* *over* *n* *random samples* *S*, *for all* 1 ≤ *r* ≤ *n*, *if we project data on the space* ${\hat{U}}_{r}$, *the expected squared residual is bounded by*

$$\sum_{j=r+1}^{n}\lambda_{j}\leq E\left[{\|P_{{{\hat{U}}_{r}}^{⊥}}\|}^{2}\right]\leq \min_{1\leq l\leq r}\left[\frac{1}{n}\sum_{j=l+1}^{n}\frac{{\hat{\theta }}_{j}\left(S\right)}{1-\gamma \;{\hat{\theta }}_{j}\left(S\right)}+\frac{1+\sqrt{l}}{\sqrt{n}}\sqrt{\frac{2}{n}\sum_{i=1}^{n}K_{ii}^{{}^{\prime }2}}\right]+R^{2}\sqrt{\frac{18}{n}\ln\left(\frac{2n}{\delta }\right)}$$ (15)

*and*

$$\sum_{j=1}^{r}\lambda_{j}\leq E\left[{\|P_{{\hat{U}}_{r}}\|}^{2}\right]\leq \max_{1\leq l\leq r}\left[\frac{1}{n}\sum_{j=1}^{l}\frac{{\hat{\theta }}_{j}\left(S\right)}{1-\gamma \;{\hat{\theta }}_{j}\left(S\right)}-\frac{1+\sqrt{l}}{\sqrt{n}}\sqrt{\frac{2}{n}\sum_{i=1}^{n}K_{ii}^{{}^{\prime }2}}\right]-R^{2}\sqrt{\frac{19}{n}\ln\left(\frac{2(n+1)}{\delta }\right)},$$ (16)

*where the support of the distribution is in a ball of radius* *R* *in the feature space*, *θ*_*i*_ *and* ${\hat{\theta }}_{i}$ *are the process and empirical eigenvalues*.

In either case, the lower bound confirms that a good representation of the data can be achieved by using the first *r* eigenvectors if the empirical eigenvalues quickly decrease before $\sqrt{l/n}$ becomes large, while the upper bound suggests that a good approximation is achievable for values of *r* where $\sqrt{r/n}$ is small. These results can be used as a benchmark to test different approaches or to select among possible candidate kernels. Also, note that depending on how much non-diagonal is K* (or **K**′), i.e. how large are the manifold mis-alignments, the KEMA bounds may be tighter than those of KPCA. With an appropriate estimation of the manifold structures via the graph Laplacians and tuning of the kernel parameters, the performance of KEMA will be at least as fitted as that of KPCA. Note that when intense regularization is needed, the trace of the squared **K**′ can be upper bounded by $\frac{1}{n\gamma^{2}}$ and then the expected squared residuals are mainly governed by *n* and *γ*.

## Results and discussion

We analyze the behavior of KEMA in a series of artificial datasets of controlled level of distortion and mis-alignment, and on real domain adaptation problems of visual object recognition from multi-source commercial databases and recognition of multi-subject facial expressions.

### Toy examples with controlled distortions and manifold mis-alignments

#### Setup

**Fig 3:**
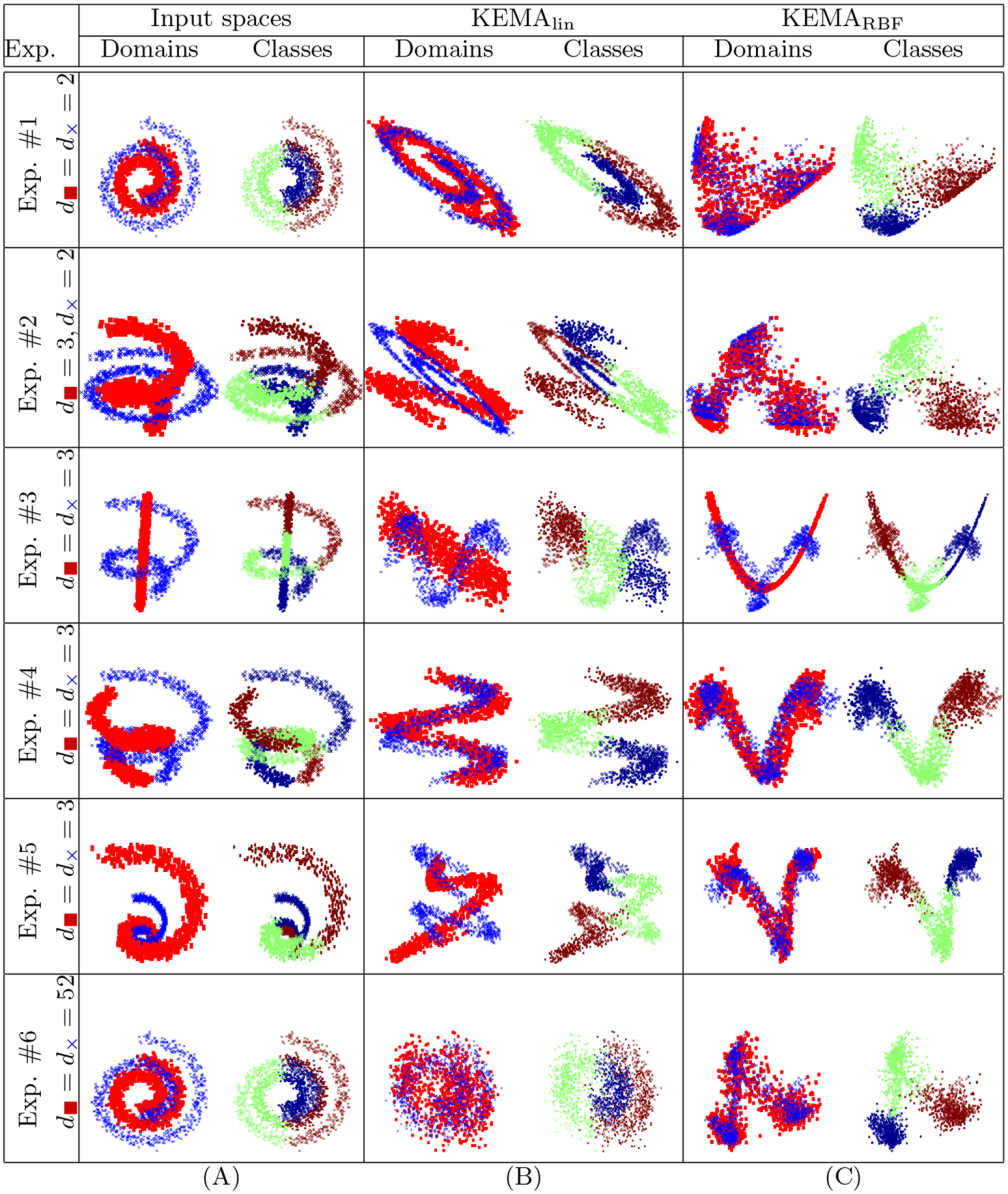
Illustration of linear and kernel manifold alignment on the toy experiments. (A) data in the original domains (X1 is designated with red squares, X2 is designated with blue crosses) and *per* class (red, green and blue circles, respectively), data projected (B) with the linear and (C) the RBF kernels.

the first set of experiments considers a series of toy examples composed of two domains with data matrices **X**_1_ and **X**_2_, which are spirals with three classes (see the two first columns of Fig 3). Each dataset is visualized by

- **domain** (first column of Fig 3): the first domain is characterized by a red square marker and the second by a blue cross. With this plot, we see if the domains are misaligned, irrespectively of the classes.
- **class** (second column of Fig 3): in this case, both domains are characterized by the class colors (red, green and blue circles). With this plot we see if the classes are aligned, irrespectively of the domain.

Then, a series of deformations are applied to the second domain: scaling, rotation, inversion of the order of the classes, the shape of the domain (spiral or line) or the data dimensionality (see Table 2). These experiments are designed to study the flexibility of KEMA to handle alignment problems of increasing complexity and between data of different dimensionality (Ex. #2). The last experiment (#6) considers the same setting of Exp. #1, but adds 50 features of Gaussian noise to the two informative features.

**Table 2:** Specification of the toy examples.

Exp.	Dimension		Deformations				Noisy
	*S*	*T*	Shape of *S*	Scaling	Rotation	Class flip	dimensions
#1	2	2	Spiral	√	-	-	0
#2	3	2	Spiral	-	-	-	0
#3	3	3	Line	-	-	-	0
#4	3	3	Spiral	-	√	√	0
#5	3	3	Spiral	√	-	√	0
#6	52	52	Spiral	√	-	-	50

For each experiment, 60 labeled pixels *per* class were sampled in each domain, as well as 1000 unlabeled samples that were randomly selected. Classification performance was assessed on 1000 held-out samples from each domain. The toy classification results can be reproduced using the MATLAB toolbox available at https://github.com/dtuia/KEMA.git. The *σ* bandwidth parameter of the RBF kernel was set in each domain as half of the median distance between all the samples in the domain, thus enforcing a domain-specific metric in each domain.

#### Latent space and domain adaptation

Fig 3 illustrates the projections obtained by KEMA when using a linear and an RBF kernel (lengthscale was set as the average distance between labeled samples). Looking at the alignment results, we observe that the linear KEMA_lin_ aligns effectively the domains only in experiments #1 and #4, which are basically scalings and rotations of the data. However, it fails on experiments #2, #3 and #5, where the manifolds have undergone stronger deformations. The use of a nonlinear kernel (KEMA_RBF_) allows much more flexible solution, performing a discriminative transform plus alignment in all experiments. In Experiment #6, even though the two discriminative dimensions (out of 52) are the same as in Exp. #1, only KEMA_RBF_ can align the data effectively, since KEMA_lin_ is strongly affected by the noise and returns a non-discriminative alignment for the eigenvectors corresponding to the smallest eigenvalues.

#### Classification performances

**Fig 4:**
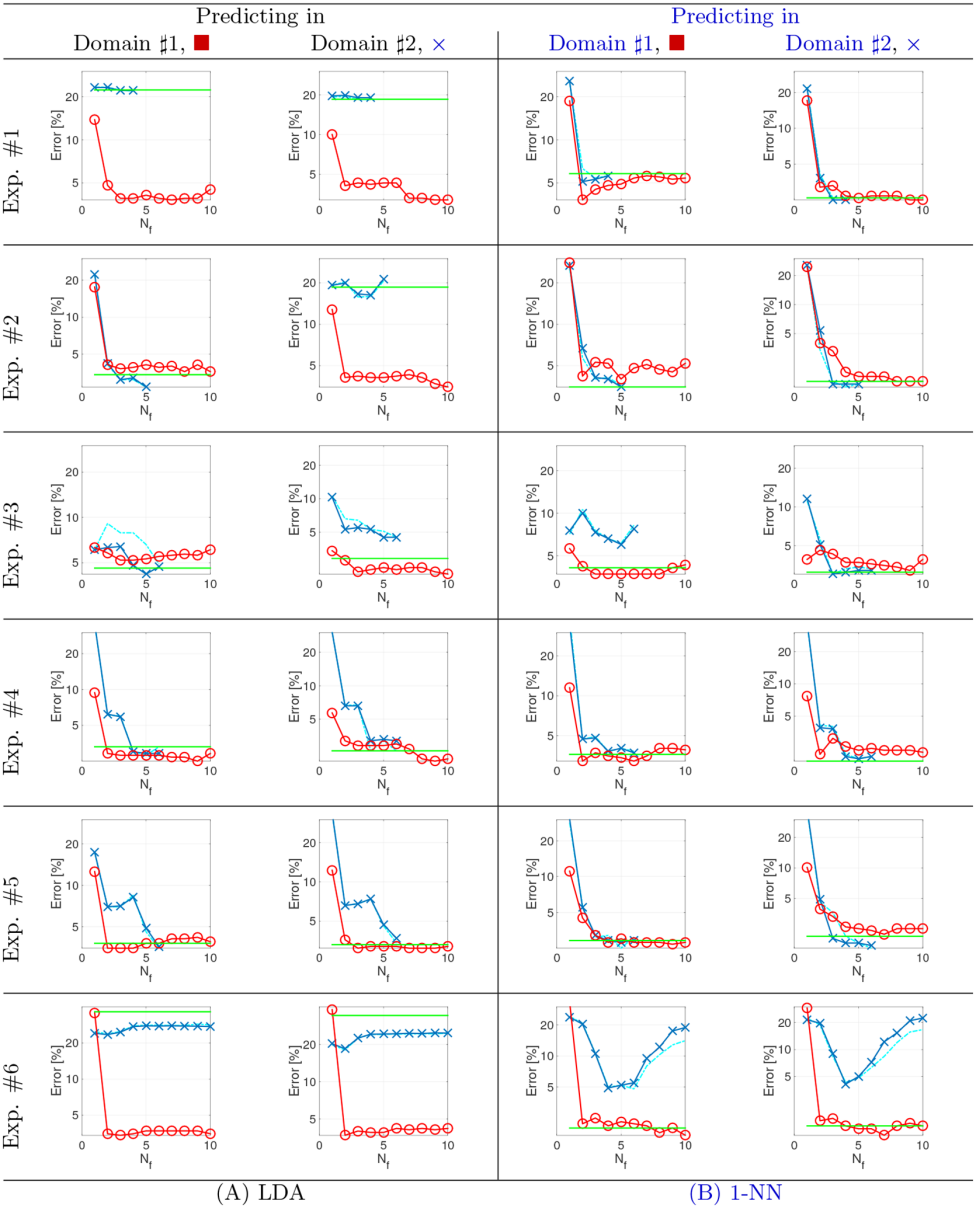
Classification performances on the toy examples. Error rates as a function of the extracted features (*N*_*f*_) when predicting data for the first (left inset) or the second (right inset) domain. In all plots KEMA_Lin_ is in blue, KEMA_RBF_ in red, SSMA in cyan and the Baseline in green. Panel (A) shows the LDA results, panel (B) the 1-NN.

Fig 4 reports the classification errors obtained by a linear discriminant analysis (LDA, Fig 4A) and the nearest neighbor classifier (1-NN, Fig 4B). For each classifier, classification errors are reported for the samples from the source domain (left inset) and the target domain (right inset). LDA is used to show the ability of projecting the domains in a joint discriminative latent space, where even the simplest linear classifier can be successful. 1-NN is used to show the increase in performance that can be obtained by using a nonlinear, yet simple, classifier on top of the projected data.

When using a linear model (LDA), a large improvement of KEMA_RBF_ over KEMA_lin_ (thus over SSMA) is observed. In experiment #1, even if the alignment is correct (Fig 3), the linear classifier trained on the projections of KEMA_lin_ cannot resolve the classification of the two domains, while KEMA_RBF_ solution provides a latent space where both domains can be classified correctly. Experiment #2 shows a different picture: the baseline error (green line in Fig 4) is much smaller in the source domain, since the dataset in 3D is linearly separable. Even if the classification of this first domain (red square in Fig 3) is correct for all methods, classification after SSMA/KEMA_lin_ projection of the second domain (blue x in Fig 3) is poor, since their projection in the latent space does not “unfold” the blue spiral. KEMA_RBF_ provides the best result. For experiment #3, the same trend as in experiment #2 is observed. Experiments #4 and #5 deal with reversed classes (the brown class is the top one in the source domain and the bottom one in the target domain). In both experiments, we observe a very accurate baseline (both domains are linearly separable in their own input spaces), but only KEMA_RBF_ provides the correct match in a low-dimensional latent space (2 dimensions), including a discriminative V-shaped projection leading to nearly 0% errors on average; KEMA_lin_ requires 5 dimensions to achieve a correct manifold alignment and a classification as accurate as the baseline (that still includes misclassifications in the linear classifier). The missclassifications can be explained by the projected space (3rd and 4th columns in Fig 3), where classes are aligned at best, but no real matching of the two data clouds is performed. The last experiment (#6) deals with noisy data, where only two out of the 52 dimensions are discriminative: KEMA_RBF_ finds the two first eigenvectors that align the data accurately (classification errors close to 0% in both domains), while KEMA_lin_ shows a much noisier alignment that, due to the rigidity of a linear transform, leads to about 20% misclassification in both domains.

When using the nonlinear 1-NN, both the KEMA_RBF_ and KEMA_lin_ perform similarly. KEMA_RBF_ still leads to correct classification with close to zero errors in all cases, thus confirming that the latent space projects samples of the same class close. KEMA_lin_ leads to correct classification in almost all the cases, since the 1-NN can cope with multimodal class distributions and nonlinear patterns in the latent space. KEMA_lin_ still fails in Exp #3, where the projection of the source domain (red circle in Fig 3) stretches over the target domain, and in Exp. # 6, where the latent space is not discriminative and harms the performance of the 1-NN.

#### Alignment with REKEMA

**Fig 5:**
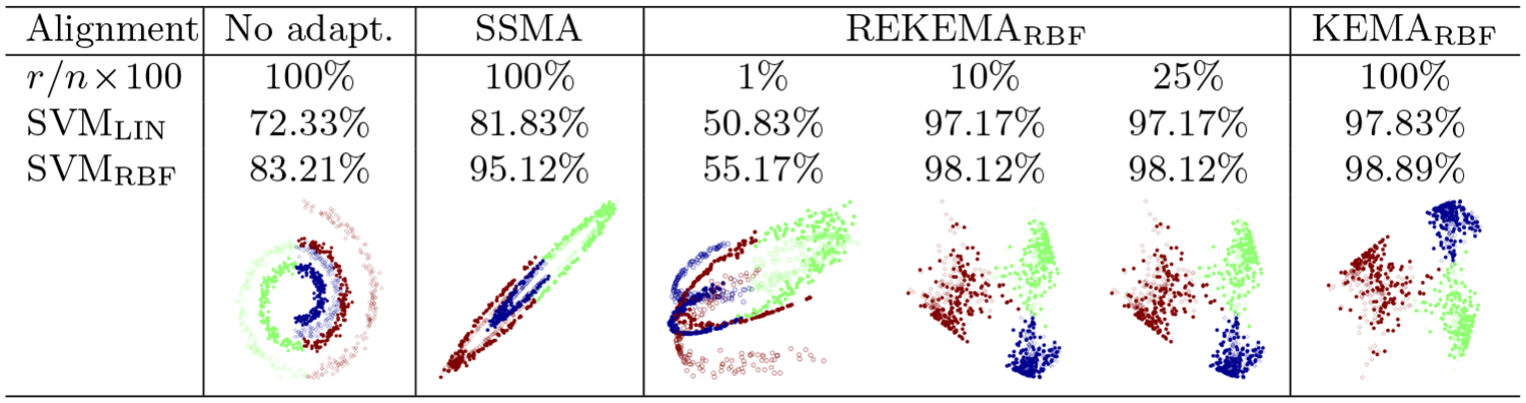
Linear and kernel manifold alignment on the scaled interwined spirals toy experiment (Exp. #1 in Fig 3). REKEMA is compared to SSMA for different rates of training samples (we used *l*_*i*_ = 100 and *u*_*i*_ = 50 per class for both domains).

We now consider the reduced-rank approximation of KEMA proposed. We used the data in the experiment #1 above. Fig 5 illustrates the solutions of the standard SSMA (or KEMA_lin_), and for REKEMA using a varying rate of samples. We also give the classification accuracies of a SVM (with both a linear and an RBF kernel) in the projected latent space. Samples were randomly chosen and the sigma parameter for the RBF kernel in KEMA_RBF_ was fixed to the average distance between all used labeled samples. We can observe that SSMA successfully aligns the two domains, but we still need to resort to nonlinear classification to achieve good results. REKEMA, on the contrary, essentially does two operations simultaneously: aligns the manifolds and increases class separability. Excessive sparsification leads to poor results. Virtually no difference between the full and the reduced-rank solutions are obtained for small values of *r*: just 10% of examples are actually needed to saturate accuracies. The proposed rKEMA showed similar behaviour but results are omitted for the sake of simplicity.

#### Invertibility of the projections

**Fig 6:**
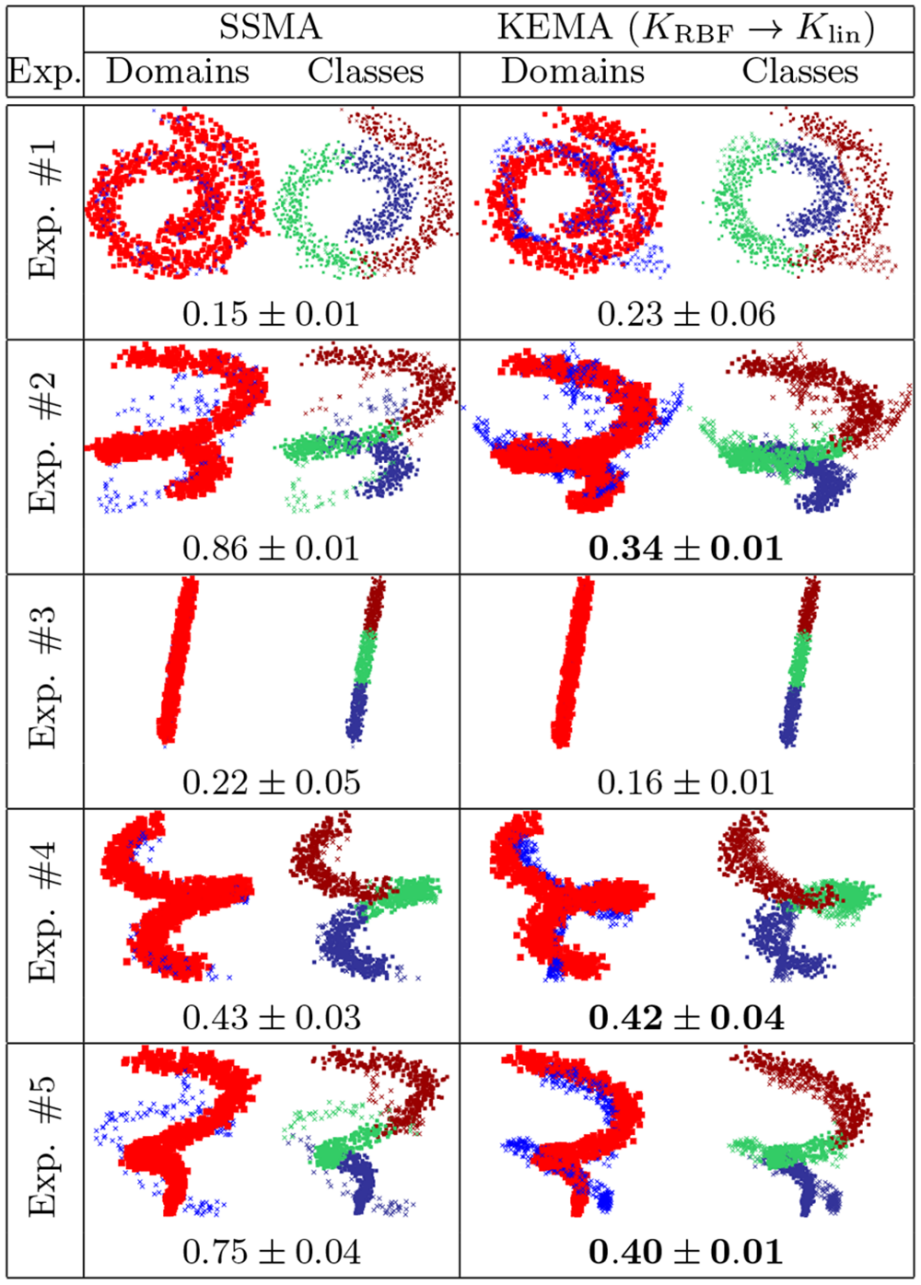
Domain inversion with SSMA and KEMA. For each panel, the left inset represents domains: the red squares are samples in the source domain, while the blue crosses are target domain samples projected onto the source domain. The right inset represents the three classes (red, green and blue circles). Each plot shows the result of a single run, and the averaged *ℓ*_2_-norm reconstruction error over 10 runs.

Fig 6 shows the results of invertibility of SSMA and KEMA (using Eq (9)) on the previous toy examples (we excluded Exp. # 6 to avoid synthesizing data with 50 noisy dimensions). We use a linear kernel for the inversion part (latent-to-source) and use for the direct part (target-to-latent space) an RBF kernel. All results are shown in the source domain space. All the other settings (# labeled and unlabeled, *μ*, graphs) are kept as in the experiments shown in Fig 3. The reconstruction error, averaged on 10 runs, is also reported: KEMA_RBF → lin_ is capable of inverting the projections and is always as accurate as the SSMA method in the simplest cases (#1, #4). For the cases related to higher levels of deformation, KEMA is either as accurate as SSMA (#3, where the inversion is basically a projection on a line) or significantly better: for experiment #2, where the two domain are strongly deformed, and experiment #5, where we deal with both scaling and inverted classes, only KEMA_RBF → lin_ can achieve satisfying inversion, as it “unfolds” the target domain and then only needs a rotation to match the distribution in the source domain.

### Visual object recognition in multi-modal datasets

We here evaluate KEMA on visual object recognition tasks by using the *Office-Caltech* dataset introduced in [2]. We consider the four domains Webcam (W), Caltech (C), Amazon (A) and DSLR (D), and selected the 10 common classes in the four datasets following [13]. By doing so, the domains contain 295 (Webcam), 1123 (Caltech), 958 (Amazon) and 157 (DSLR) images, respectively. The features were extracted in two ways

- SURF features, as described in [2]: we use a 800-dimensional normalized histogram of visual words obtained from a codebook constructed from a subset of the Amazon dataset on points of interest detected by the Speeded Up Robust Features (SURF) method. The features are included in the in the MATLAB package on https://github.com/dtuia/KEMA.git. Alternatively, they can be downloaded from their original repository on https://www.eecs.berkeley.edu/jhoffman/domainadapt/.
- Deep features from DeCAF [43]: these features are extracted as the sparse activations of the fully connected 7th layer of of a convolutional network trained on imageNet and then fine tuned on the visual recognition tasks considered here. It forms a 4096-dimensional vector. The features are included in the MATLAB package on https://github.com/dtuia/KEMA.git.

#### Experimental setup

We compare our proposed KEMA with the following unsupervised and semi-supervised domain adaptation methods: GFK [13], OT-lab [15] and JDA [26]. We used the same experimental setting as [13], in order to compare with these unsupervised domain adaptation methods. For all methods, we used 20 labeled pixels *per* class in the source domain for the C, A and W domains and 8 samples *per* class for the D domain. After alignment, an ordinary 1-NN classifier was trained with the labeled samples. The same labeled samples in the source domain were used to define the PLS eigenvectors for GFK and OT-lab. For all the methods using labeled samples in the target domain (including KEMA), we used 3 labeled samples in target domain to define the projections.

We used a sensible kernel for this problem in KEMA: the (fast) histogram intersection kernel [44]. Using a *χ*_2_ kernel resulted in similar performances. We used *u* = 300 unlabeled samples to compute the graph Laplacians, for which a *k*-NN graph with *k* = 21 was used.

#### Numerical results

**Fig 7:**
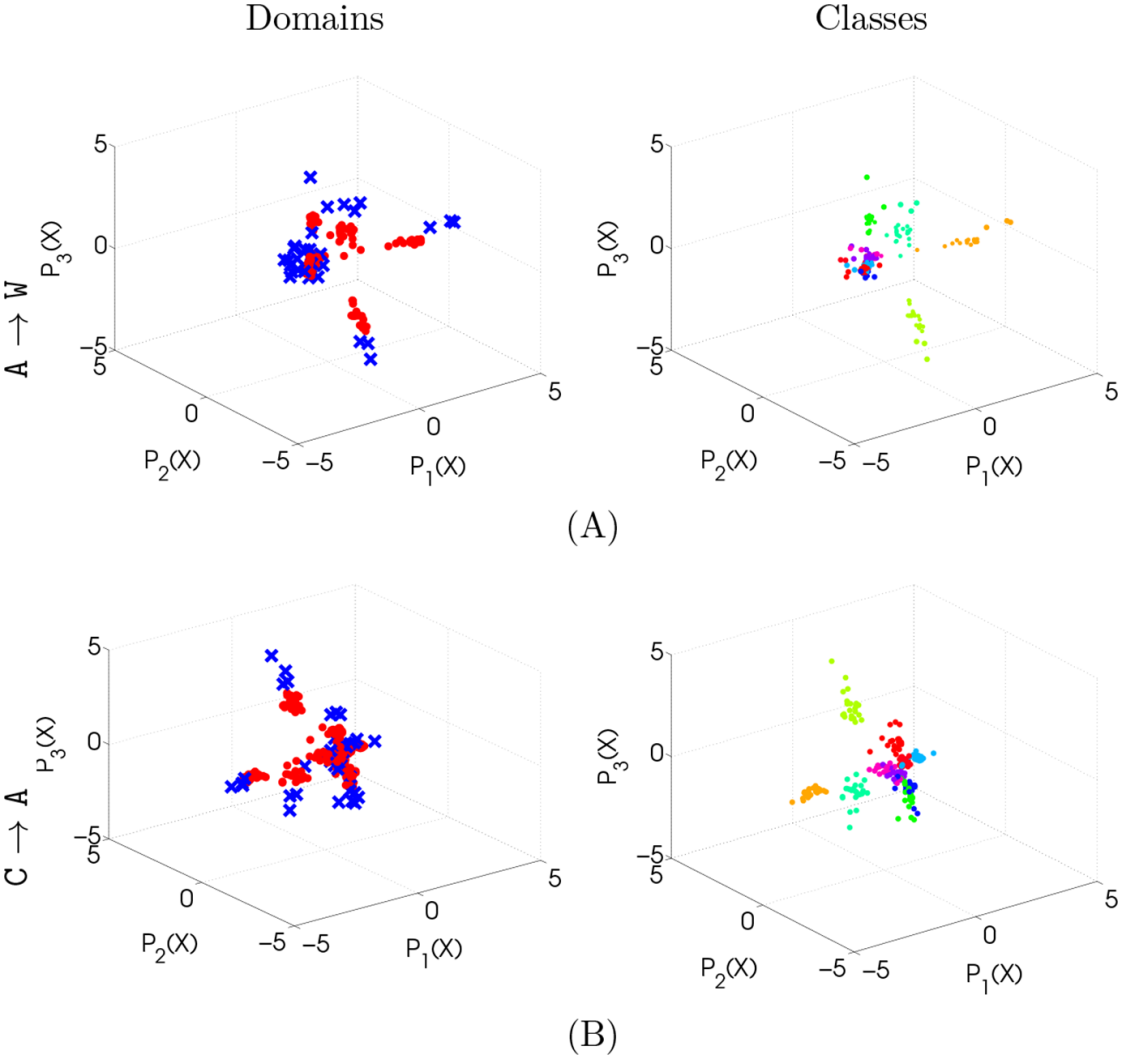
Example of the three first dimensions of the latent space. (A) illustrates the A → W experiment. (B) illustrates the C → A experiment. Left: by domain (red circles are the source samples, blue crosses are the target samples), right: by class (each color represents a different class).

The projections obtained by KEMA in the visual object recognition experiments remain discriminative, as shown by Fig 7, where projections on the first three dimensions of the latent space are reported for the A → W (top) and C → A (bottom) using the SURF features. The numerical results obtained in all the eight problems are reported in Table 3: KEMA outperforms the unsupervised GFK and, in most of the cases, improves the results obtained by the semi-supervised methods using labels in the source domain only. KEMA provides the most accurate results in 5 out of the 8 settings. KEMA is as accurate as the state of the art, but with the advantage of handling naturally domains of different dimensionality, and not requiring semilabeled examples ($\hat{y}$ in the Table) to align the domains as JDA. The results obtained when using the deep DeCAF features are reported in Table 4: a strong improvement in performance is observed for all methods. This general increase was expected, since the deep features in DeCAF are naturally suited for domain adaptation (they are extracted with fine tuning on this specific dataset): but nonetheless, even if the boost in performance is visible for all the methods (including the case without adaptation), KEMA improves performances even further and leads to the best average results. Looking at the single experiments, KEMA performs most often on a tie with OT-lab [15]. Summing up, KEMA leads to results as accurate as the state of art, but is much more versatile, since it allows to handle unpaired data, works with datasets of different dimensionality, and has a significantly smaller computational load (see also Table 1 for a taxonomical comparison of the properties of the different methods).

**Table 3:** 1-NN classification accuracy in the visual object recognition study using the SURF features.

	Train on source No adapt.	Unsup. GFK [13]	DA Labels: *S*		Labels: *S*, *T* KEMA *K*_int_	Train on target No adapt.
			OT-lab [15]	JDA [26]		
*l*_*S*_		0	20	20	20	
*l*_*T*_		0	0	$\hat{y}$	3	
C → A	21.4±3.7	35.3±3.2	43.5±2.1	40.7±4.0	**47.1** **±** **3.0**	35.4±2.4
C → D	12.3±2.8	35.6±5.0	41.8±2.8	40.0±4.0	**61.5** **±** **2.8**	65.1±1.9
A → C	35.3±0.5	32.9±2.5	**35.2** **±** **0.8**	34.0±3.1	29.5±3.0	28.4±1.6
A → W	31.0±0.7	32.0±3.4	38.4±5.4	36.0±5.1	**65.4** **±** **2.7**	63.5±2.6
W → C	21.7±0.4	27.7±2.4	**35.5** **±** **0.9**	31.8±1.9	32.9±3.3	28.4±1.6
W → A	27.0±1.5	33.3±2.1	40.0±1.0	31.5±4.7	**44.9** **±** **4.5**	35.4±2.4
D → A	19.0±2.2	33.0±1.3	34.9±1.3	32.9±2.9	**44.2** **±** **3.1**	35.4±2.4
D → W	37.4±3.0	69.7±3.8	**84.2** **±** **1.0**	80.0±4.1	64.1±2.9	63.5±2.6
**Mean**	22.3	37.4	44.2	40.9	**48.7**	44.4

C: Caltech, A: Amazon, D: DSLR, W: Webcam.

*l*_domain_: number of labels per class.

$\hat{y}$: predicted labels.

**Table 4:** 1-NN classification accuracy in the visual object recognition study using the DeCAF fully connected layer fc7.

fc7	Train on source No adapt.	Unsup. GFK [13]	DA labels: *S*		labels: *S*, *T* KEMA *K*_int_	Train on target No adapt.
			OT-lab [15]	JDA [26]		
*l*_*S*_		0	20	20	20	
*l*_*T*_		0	0	$\hat{y}$	3	
C → A	84.5±1.5	87.8±2.1	**92.1** **±** **1.3**	89.6±2.0	91.5±1.5	84.4±3.6
C → D	73.1±4.9	83.5±3.6	85.4±6.0	85.0±4.9	**93.6** **±** **3.1**	92.2±1.9
A → C	72.0±1.7	80.2±1.9	**87.2** **±** **1.2**	82.6±2.9	80.3±3.4	66.3±3.7
A → W	61.3±3.4	78.0±4.8	84.5±2.4	83.0±4.6	**92.7** **±** **2.5**	88.1±3.8
W → C	68.9±3.0	75.1±2.5	**83.7** **±** **1.5**	79.8±2.0	82.1±2.3	66.3±3.7
W → A	73.5±2.7	81.2±2.2	**91.9** **±** **1.4**	90.9±1.2	91.6±1.3	84.4±3.6
D → A	74.6±3.9	85.4±2.1	**92.9** **±** **1.1**	91.9±0.8	90.3±1.1	84.4±3.6
D → W	93.8±1.5	96.7±1.9	**94.1** **±** **3.4**	97.0±1.5	91.0±3.5	88.1±3.8
**Mean**	75.2	83.5	88.9	87.49	**89.1**	81.7

C: Caltech, A: Amazon, D: DSLR, W: Webcam.

*l*_domain_: number of labels per class.

$\hat{y}$: predicted labels.

### Recognition of facial expressions in multi-subject databases

This experiment deals with the task of recognizing facial expressions. We used the dataset in [45], where 185 photos of three subjects depicting three facial expressions (happy, neutral and shocked) are available. The features are included in the MATLAB package on https://github.com/dtuia/KEMA.git. Alternatively, they can be downloaded from their original repository on http://www.cc.gatech.edu/lsong/code.html. Each image is 217 × 308 pixels and we take each pixel as one dimension for classification (66836 dimensional problem). Each pair {subject,expression} has around 20 repetitions.

#### Experimental setup

Different subjects represent the domains and we align them with respect to the three expression classes. We used only three labeled examples *per* class and subject, and held out 70% of the data for testing and used the remaining 30% (55 samples) for the extraction of the labeled samples. The examples which have not been selected as labeled points are used as unlabeled data. The three domains are aligned simultaneously into a common latent space, and then all classifications are run therein for all subjects. Below, we report the results obtained by using a LDA classifier trained in that common latent space. We consider three experimental settings:

- Single resolution: all images are considered at their maximal resolution accounting for the three domains. Each domain is therefore a 66836-dimensional dataset. SSMA could not handle these data, since it would involve a 200508-dimensional eigendecomposition.
- Multiresolution, factor 2: the resolution of one of the domains (Subject #1) is downgraded by a factor two. 154 × 109, leading to a 16786-dimensional domain. The alignment problem in the primal would then be 16786 + (2 × 66836) = 150458-dimensional. With this experiment, we aim at showing the capability of KEMA to handle data of different dimensionality.
- Multiresolution, factor 4: the resolution of one of the domains (Subject #1) is downgraded by a factor four. 62 × 44, leading to a 2728-dimensional domain. The alignment problem in the primal would then be 136400-dimensional.

#### Numerical results

**Fig 8:**
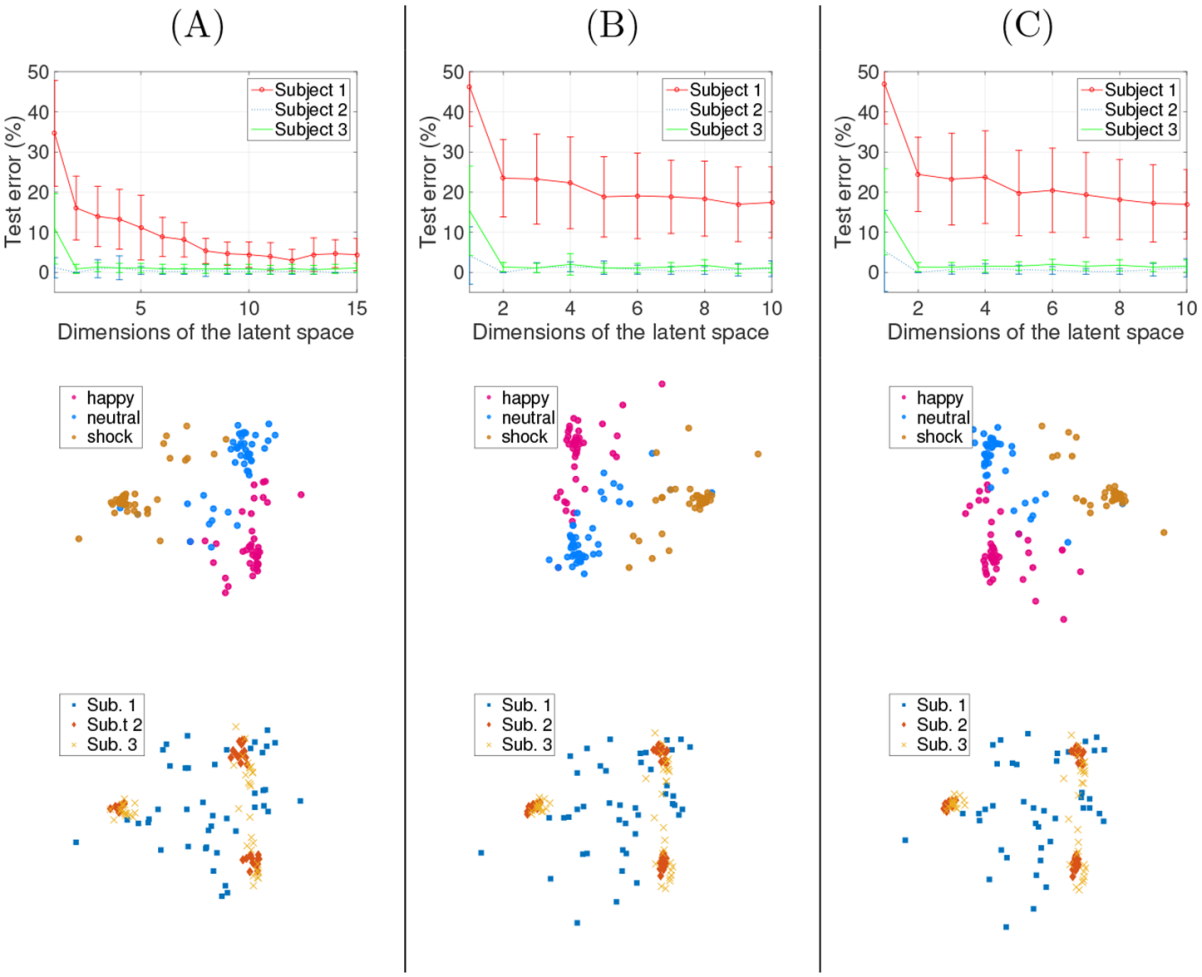
Results of the classification of facial expressions (top: error rates, middle: predicted expressions; bottom: subjects). (A) single resolution experiment; (B) multiresolution experiment with a factor-two reduction for the images of subject 1; (C) multiresolution experiment with a factor-four reduction for the images of subject 1.

Average results over ten realizations are given in Fig 8: since it works directly in the dual, KEMA can effectively cast the three-domains problem into a low dimensional space. In the single resolution case (Fig 8B) all domains are classified with less than 5% error. This shows an additional advantage of KEMA with respect to SSMA in high dimensional spaces: SSMA would have required to solve a 200508-dimensional eigenproblem, while KEMA solves only a 55-dimensional problem. Subject #1 seems to be the most difficult to align with the two others, difficulty that is also reflected in the higher classification errors. Actually, subject #1 shows little variations in his facial traits from one expression to the other compared to the other subjects (see Fig 3 in [45]).

In the multi-resolution cases, similar error rates are observed for subjects #2 and #3, even though the images of subject #1 were of coarse resolution. The reduced resolution of the images of subject #1 made the expression recognition harder, but error rates lower than 20% are still achieved by using KEMA. By looking at the projections (second and third rows of Fig 8), those of the multiresolution experiment with a factor 2 reduction ((B) panel) are very similar to those in the single resolution experiment ((A) panel).

## Conclusions

We introduced a kernel method for semi-supervised manifold alignment. We want to stress that this particular kernelization goes beyond the standard academic exercise as the method addresses many problems in the literature of domain adaptation and manifold learning. The so-called KEMA can actually align an arbitrary number of domains of different dimensionality without needing corresponding pairs, just few labeled examples in all domains. We also showed that KEMA generalizes SSMA when using a linear kernel, which allows us to deal with high-dimensional data efficiently in the dual form. Working in the dual can be computationally costly because of the construction of the graph Laplacians and the size of the involved kernel matrices. Regarding the Laplacians, they can be computed just once and off-line, while regarding the size of the kernels, we introduced a reduced-ranked version that allows to work with a fraction of the samples while maintaining the accuracy of the representation. Advantageously, KEMA can align manifolds of very different structures and dimensionality, performing a discriminative transform along with the alignment. We have also provided a simple yet effective way to map data between domains as an alternative to standard pre-imaging techniques in the kernel methods literature. This is an important feature that allows synthesis applications, but more remarkably allows to study and characterize the distortion of the manifolds in physically meaningful units. To the authors’ knowledge this is the first method in addressing all these important issues at once. All these features were illustrated through toy examples of increasing complexity (including data of different dimensionality, noise, warps and strong nonlinearities) and real problems in computer vision, and face recognition, thus showing the versatility of the method and its interest for numerous application domains. It does not escape our attention that KEMA may become a standard multivariate method for data preprocessing in general applications where multisensor, multimodal, sensory data is acquired.

## Figures and Tables

**Fig 9:**
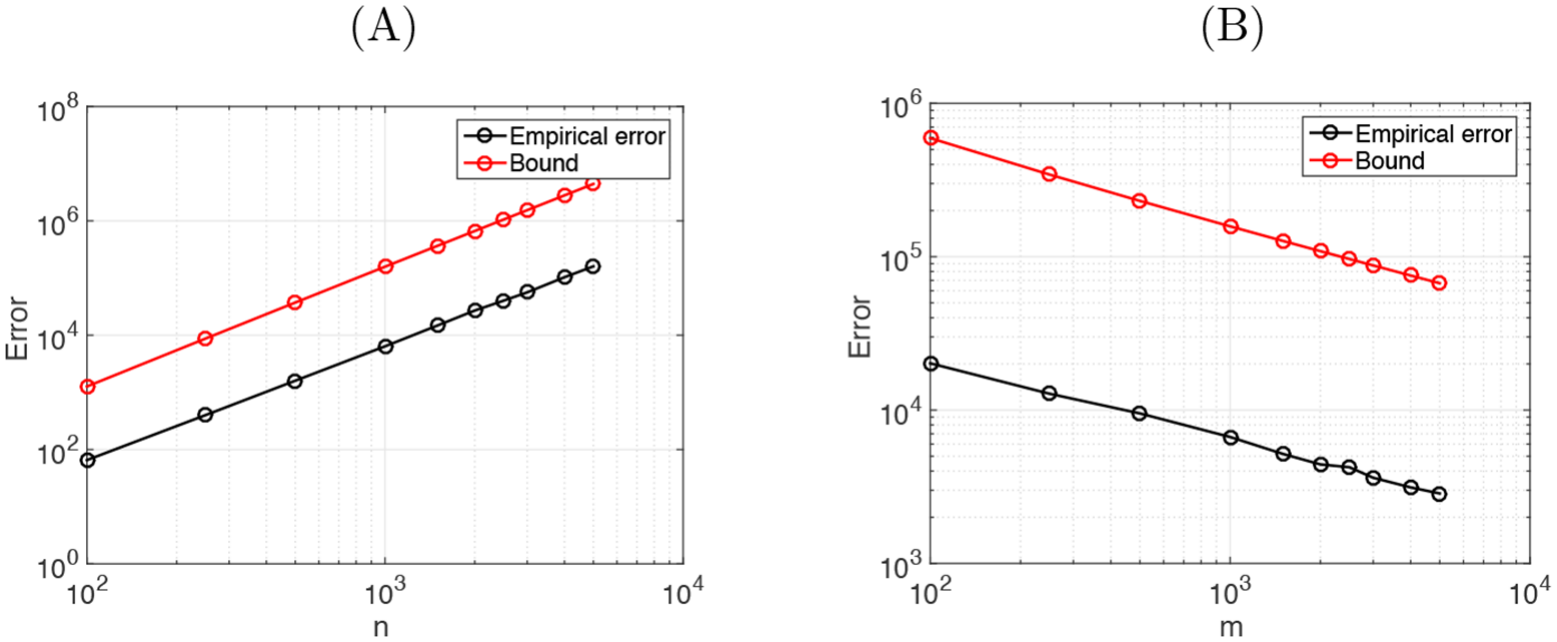
Error of the approximation, $\text{Error}≔\|{\hat{K}}_{d}^{-1}{\hat{K}}_{s}-K_{d}^{-1}K_{s}{\|}_{F}$ as a function of the number of samples n (left) and number of random features m (right) used in the approximation of the eigensystem equation of KEMA.

## Appendix: Convergence bounds for rKEMA

In this Appendix, we study some theoretical properties of the proposed *randomized* KEMA (rKEMA, page 9) to provide some guarantees of its convergence to KEMA. The solution of KEMA is the eigensystem of the matrix $K_{d}^{-1}K_{s}$ —or alternatively its Tikhonov-regularized problem (**K**_*d*_ + γ**I**)^−1^ **K**_*s*_. The matrices are now approximated by ${\hat{K}}_{s}=Z_{s}Z_{s}^{⊤}$ and ${\hat{K}}_{d}=Z_{d}Z_{d}^{⊤}$, see Eq (11). Our aim is to give a bound on the approximation error to a product of these two matrices from products of their approximations through random projection matrices. First we recall the Hermitian Matrix Bernstein theorem, which is then used to derive the bound on rKEMA.

**Theorem 4 (Matrix Bernstein, [46])** *Let* **Z**_1_, …, **Z**_*m*_ *be independent* *n* × *n* *random matrices. Assume that* $E[Z_{i}]=0$ *and that the norm of the error matrices is bounded* ‖**Z**_*i*_‖ ≤ *R*. *Define the variance parameter* $\sigma^{2}≔\max\{\|\sum_{i}E\left[Z_{i}^{⊤}Z_{i}\right]\|,\|\sum_{i}E\left[Z_{i}Z_{i}^{⊤}\right]\|\}$. *Then, for all* *t* ≥ 0,

$$\mathbb{P}\left(\left\|\underset{i}{\sum }Z_{i}\geq t\right\|\right)\leq 2n\exp\left(\frac{-t^{2}}{3\sigma^{2}+2Rt}\right)\;and\;E\left\|\underset{i}{\sum }Z_{i}\right\|\leq \sqrt{3\sigma^{2}\log\left(n\right)}+R\log\left(n\right).$$ (17)

**Theorem 5** *Given two kernel matrices* **K**_*d*_ *and* **K**_*s*_, *we aim to solve the eigensystem of* $K_{d}^{-1}K_{s}$. *Let us define the corresponding kernel approximations* ${\hat{K}}_{d}$, ${\hat{K}}_{s}$ *using* *m*_*d*_, *m*_*s*_ *random features as in* Eq (12), *respectively, and* *m*: = min(*m*_*d*_, *m*_*s*_). *Then, the* *ℓ*_2_ *approximation error bound can be bounded as*

$$E\|{\hat{K}}_{d}^{-1}{\hat{K}}_{s}-K_{d}^{-1}K_{s}\|\leq \sqrt{\frac{3n^{4}\log\left(n\right)}{m}}+\frac{2n^{2}\log\left(n\right)}{m}.$$ (18)

**Proof 1** *For the sake of simplicity, let us rename* $\hat{D}={\hat{K}}_{d}^{-1}$ *and* $D=K_{d}^{-1}$. *We follow a similar derivation to* [47] *for randomized nonlinear CCA. The total error matrix can be decomposed as a sum of individual error terms*, $E=\sum_{i=1}^{m_{s}}E_{i}$, *which are defined as* $E_{i}=\frac{1}{m_{s}}(\hat{D}{\hat{K}}_{s}^{\left(i\right)}-DK_{s})$. *Now recall that the* *m*_*d*_+*m*_*s*_ *random features are sampled i.i.d. and that the data matrices for each domain are constant. Therefore, the random matrices* $\left\{{\hat{D}}^{\left(1\right)},\ldots ,{\hat{D}}^{\left(m_{d}\right)},{\hat{K}}_{s}^{\left(1\right)},\ldots ,{\hat{K}}_{s}^{\left(m_{s}\right)}\right\}$ *are i.i.d. random variables. Hence, their expectations factorize*, $E\;[E_{i}]=\frac{1}{m_{s}}\;(E\left[\hat{D}\right]K_{s}-DK_{s})$, *where we used* $E\left[{\hat{K}}_{s}^{\left(i\right)}\right]=K_{s}$. *The deviation of the individual error matrices from their expectations is* $Z_{i}=E_{i}-E\;\left[E_{i}\right]=\frac{1}{m_{s}}(\hat{D}{\hat{K}}_{s}^{\left(i\right)}-E\left[\hat{D}\right]K_{s})$. *Now we can apply Hölder’s condition twice after using the triangle inequality on the norm, and Jensen’s inequality on the expected values and obtain a bound of the error matrices*, *R*:

$$\|Z_{i}\|=\frac{1}{m_{s}}\left\|\hat{D}{\hat{K}}_{s}^{\left(i\right)}-E\left[\hat{D}\right]K_{s}\right\|\leq \frac{B(B+\|K_{s}\|)}{m_{s}},$$ (19)

*where* *B* *is a bound on the norm of the randomized feature map*, ‖**z**‖^2^ ≤ *B*. *The variance is defined as* $\sigma^{2}≔\max\{\|\sum_{i=1}^{m_{s}}E\left[Z_{i}Z_{i}^{⊤}\right]\|,\|\sum_{i=1}^{m_{s}}E\left[Z_{i}^{⊤}Z_{i}\right]\|\}$. *Let us expand the individual terms in the (first) summand*:

$$\begin{array}{c} Z_{i}^{⊤}Z_{i}=\frac{1}{m_{s}^{2}}\left({\hat{K}}_{s}^{\left(i\right)}{\hat{D}}^{2}{\hat{K}}_{s}^{\left(i\right)}+K_{s}E{\left[\hat{D}\right]}^{2}K_{s}-{\hat{K}}_{s}^{\left(i\right)}\hat{D}E\left[\hat{D}\right]K_{s}-E\left[\hat{D}\right]K_{s}\hat{D}{\hat{K}}_{s}^{\left(i\right)}\right), \end{array}$$ (20)

*and now taking the norm of the expectation, and using Jensen’s inequality, we obtain* $\|E[Z_{i}^{⊤}Z_{i}]\|\leq \frac{B^{2}{\left\|K_{s}\right\|}^{2}}{m^{2}}$, *which is the same for* $\left\|E[Z_{i}Z_{i}^{⊤}]\right\|$, *and therefore the worst-case estimate of the variance is* $\sigma^{2}\leq \frac{B^{2}{\left\|K_{s}\right\|}^{2}}{m_{s}}$. *The bound can be readily obtained by appealing to the matrix Bernstein inequality (Theorem 4) and using the fact that random features and kernel evaluations are upper-bounded by 1, and thus both* *B* *and* ‖**K**‖ *are upper-bounded by* *n*.

**Theorem 6** *Equivalently, when we define the corresponding bound for a Tikhonov-regularized problem as* (**K**_*d*_ + γ**I**)^−1^ **K**_*s*_, *and its approximation as* ${\left({\hat{K}}_{d}+\gamma I\right)}^{-1}{\hat{K}}_{s}$, *the bound reduces to*

$$E\|{\left({\hat{K}}_{d}+\gamma I\right)}^{-1}{\hat{K}}_{s}-{\left(K_{d}+\gamma I\right)}^{-1}K_{s}\|\leq \frac{1}{\gamma }\left(\sqrt{\frac{3n^{2}\log\left(n\right)}{m}}+\frac{2n\log(n)}{m}\right),$$ (21)

*where* *γ* > 0 *is a regularization parameter*.

**Proof 2** *The demonstration is trivial by following the same rationale and derivations in Theorem 5, and simply bounding* $\|{\left({\hat{K}}_{d}+\gamma I\right)}^{-1}{\|}^{2}\leq 1/\gamma$. *Interestingly, the bound is exactly the same as that of the randomized nonlinear CCA in* [47] *for the case of paired examples in the domains and no graph Laplacian terms*.

Fig 9 shows the absolute error committed by doing an approximation with random features of the corresponding kernels for rKEMA, along with the derived theoretical bound. We analyze the issue as a function of *m* (here for the sake of simplicity we used *m*_*d*_ = *m*_*s*_ = *m*), and the number of samples *n*. The curves are the result of 300 realizations. The reported results match the previous bound: we observe a logarithmical trend as a function of *m* (linear in the log-scale, $O(m^{-1/2})$), and *n* log(*n*) for the case of training examples, as expected.

## References

[1] Quiñonero-Candela J, Sugiyama M, Schwaighofer A, Lawrence ND. Dataset shift in machine learning Neural information processing series. Cambridge, Mass., London: MIT Press; 2009.

[2] Saenko K, Kulis B, Fritz M, Darrell T. Adapting visual category models to new domains In: Proc. ECCV. Berlin, Heidelberg: Springer-Verlag; 2010 p. 213–226.

[3] Farhadi A, Tabrizi MK. Learning to Recognize Activities from the Wrong View Point In: Proc. ECCV. Berlin, Heidelberg: Springer-Verlag; 2008 p. 154–166.

[4] Duan L, Xu D, Tsang IW, Luo J. Visual Event Recognition in Videos by Learning from Web Data. IEEE Trans Pattern Anal Mach Intell. 2012;34(9):1667–1680. 10.1109/TPAMI.2011.265 22201057

[5] Torralba A, Efros AA. Unbiased look at dataset bias In: Proc. CVPR. Colorado Springs, CO; 2011 p. 1521–1528.

[6] Pan SJ, Yang Q. A Survey on Transfer Learning. IEEE Transactions on Knowledge and Data Engineering. 2010 10;22(10):1345–1359. 10.1109/TKDE.2009.191

[7] Patel VM, Gopalan R, Li R, Chellappa R. Visual Domain Adaptation: A survey of recent advances. IEEE Signal Proc Mag. 2015 5;32(3):53–69. 10.1109/MSP.2014.2347059

[8] Jacobs DW, Daume H, Kumar A, Sharma A. Generalized Multiview Analysis: A discriminative latent space In: Proc. CVPR. Providence, RH; 2012 p. 2160–2167.

[9] Lai PL, Fyfe C. Kernel and Nonlinear Canonical Correlation Analysis. In: Int. J. Neural Sys.; 2000 p. 365–377. 10.1142/S012906570000034X 11195936

[10] Pan SJ, Yang Q. Domain adaptation via transfer component analysis. IEEE Trans Neural Networks. 2011;22:199–210. 10.1109/TNN.2010.2091281 21095864

[11] Baktashmotlagh M, Harandi MT, Lovell BC, Salzmann M. Domain adaptation on the statistical manifold In: Proc. CVPR. Columbus, OH; 2014 p. 2481–2488.

[12] Gopalan R, Li R, Chellappa R. Domain adaptation for object recognition: An unsupervised approach In: Proc. ICCV. Barcelona, Spain; 2011 p. 999–1006.

[13] Gong B, Shi Y, Sha F, Grauman K. Geodesic flow kernel for unsupervised domain adaptation In: Proc. CVPR. Providence, RH: IEEE; 2012 p. 2066–2073.

[14] Gretton A, Bousquet O, Smola AJ, Schölkopf B. Measuring statistical dependence with Hilbert-Schmidt norms. In: Jain S, Lee WS, editors. Proc. Algorithmic Learn. Theory; 2005 p. 63–77.

[15] Courty N, Flamary R, Tuia D. Domain adaptation with regularized optimal transport In: Proc. ECML. Nancy, France; 2014 p. 274–289.

[16] Kulis B, Saenko K, Darrell T. What you saw is not what you get: domain adaptation using asymmetric kernel transforms In: Proc. CVPR. Colorado Springs, CO; 2011 p. 1785–1792.

[17] Jhuo IH, Liu D, Lee DT, Chang SF. Robust visual domain adaptation with low-rank reconstruction In: Proc. CVPR. Providence, RH; 2012 p. 2168–2175.

[18] Hoffman J, Rodner E, Donahue J, Saenko K, Darrell T. Efficient Learning of Domain Invariant Image Representations In: Proc. ICLR. Scottsdale, AZ; 2013.

[19] Donahue J, Hoffman J, Rodner E, Saenko K, Darrell T. Semi-supervised Domain Adaptation with Instance Constraints. In: CVPR; 2013 p. 668–675.

[20] Ham J, Lee D, Saul L. Semisupervised alignment of manifolds In: Cowell RG, Ghahramani Z, editors. Proc. AISTATS. London, UK; 2005 p. 120–127.

[21] Wang C, Krafft P, Mahadevan S. Manifold alignment In: Ma Y, Fu Y, editors. Manifold Learning: Theory and Applications. CRC Press; 2011.

[22] Hotelling H. Relations Between Two Sets of Variates. Biometrika. 1936 12;28(3/4):321–377. 10.1093/biomet/28.3-4.321

[23] Wang C, Mahadevan S. Heterogeneous domain adaptation using manifold alignment In: IJCAI. Barcelona, Spain; 2011 p. 1541–1546.

[24] Jolliffe IT. Principal Component Analysis. New York: Springer; 1986.

[25] Schölkopf B, Smola AJ, Müller KR. Nonlinear component analysis as a kernel Eigenvalue problem. Neural Comput. 1998;10:1299–1319. 10.1162/089976698300017467

[26] Long M, Wang J, Ding G, Sun J, Yu PS. Transfer Feature Learning with Joint Distribution Adaptation. In: ICCV; 2013 p. 2200–2207.

[27] Tuia D, Muñoz-Marí J, Gómez-Chova L, Malo J. Graph matching for adaptation in remote sensing. IEEE Trans Geosci Remote Sens. 2013;51(1):329–341. 10.1109/TGRS.2012.2200045

[28] Mika S, Schölkopf B, Smola A, Müller KR, Scholz M, Rätsch G. Kernel PCA and De-Noising in Feature Spaces In: NIPS 11 MIT Press; 1999 p. 536–542.

[29] Bakιr G, Weston J, Schölkopf B. Learning to find Pre-images. In: Proc. NIPS; 2003.

[30] Kwok JT, Tsang IW. The Pre-Image Problem in Kernel Methods. IEEE Trans Neural Networks. 2004;15(6):1517–1525. 10.1109/TNN.2004.837781 15565778

[31] Chapelle O, Schölkopf B, Zien A. Semi-Supervised Learning. 1st ed Cambridge, MA and London, England: MIT Press; 2006.

[32] Camps-Valls G, Bandos T, Zhou D. Semi-supervised Graph-based Hyperspectral Image Classification. IEEE Transactions on Geoscience and Remote Sensing. 45(10):2044–3054.

[33] Tuia D, Volpi M, Trolliet M, Camps-Valls G. Semisupervised Manifold Alignment of Multimodal Remote Sensing Images. IEEE Trans Geosci Remote Sens. 2014;52(12):7708–7720. 10.1109/TGRS.2014.2317499

[34] Tuia D, Trolliet M, Volpi M. Multisource alignment of image manifolds. In: IEEE International Geoscience and Remote Sensing Symposium, IGARSS. Melbourne, Australia; 2013.

[35] Reed M, Simon B. I: Functional Analysis, Volume 1 (Methods of Modern Mathematical Physics) (vol 1). 1st ed Academic Press; 1981.

[36] Riesz F, Nagy BS. Functional Analysis. Frederick Ungar Publishing Co; 1955.

[37] Yan S, Xu D, Zhang B, Zhang HJ, Yang Q, Lin S. Graph Embedding and Extensions: A General Framework for Dimensionality Reduction. IEEE Trans Patt Anal Mach Intell. 2007;29(1):40–51. 10.1109/TPAMI.2007.250598 17108382

[38] Zhu F, P H, Kallas M. Kernel nonnegative matrix factorization without the pre-image problem In: Machine Learning for Signal Processing. Reims,France; 2014.

[39] Rahimi A, Recht B. Random Features for Large-Scale Kernel Machines. In: Neural Information Processing Systems; 2007.

[40] Jones LK. Annals of Statistics. A simple lemma on greedy approximation in Hilbert space and convergence rates for projection pursuit regression and neural network training. 1992;20:608–613.

[41] Shawe-Taylor J, Williams CKI, Cristianini N, Kandola J. On the eigenspectrum of the Gram matrix and the generalization error of kernel-PCA. IEEE Trans Info Theory. 2005;51(7):2510–2522. 10.1109/TIT.2005.850052

[42] Dhanjal C, Gunn SR, Shawe-Taylor J. Efficient Sparse Kernel Feature Extraction Based on Partial Least Squares. IEEE Trans Pattern Anal Mach Intell. 2009;31(8):1347–1361. 10.1109/TPAMI.2008.171 19542571

[43] Donahue J, Jia Y, Vinyals O, Hoffman J, Zhang N, Tzeng E, et al. DeCAF: A Deep Convolutional Activation Feature for Generic Visual Recognition. In: Proceedings of The 31st International Conference on Machine Learning; 2014. p. 647–655.

[44] Maji S, Berg AC, Malik J. Classification using intersection kernel support vector machines is efficient. In: 2008 IEEE Computer Society Conference on Computer Vision and Pattern Recognition (CVPR 2008), 24–26 June 2008, Anchorage, Alaska, USA; 2008.

[45] Song L, Smola A, Gretton A, Borgwardt KM. A Dependence Maximization View of Clustering In: Proc. ICML. Corvallis, OR; 2007 p. 815–822.

[46] Mackey L, Jordan MI, Chen RY, Farrell B, Tropp JA. Matrix Concentration Inequalities via the Method of Exchangeable Pairs. Annals of Probability. 2014;.

[47] Lopez-Paz D, Sra S, Smola AJ, Ghahramani Z, Schölkopf B. Randomized Nonlinear Component Analysis. In: Proceedings of the 31 st International Conference on Machine Learning. Beijing, China; 2014. p. 1–9.

